# Simultaneous achieving negative photoconductivity response and volatile resistive switching in Cs_2_CoCl_4_ single crystals towards artificial optoelectronic synapse

**DOI:** 10.1038/s41377-024-01642-8

**Published:** 2024-12-02

**Authors:** Huifang Jiang, Huifang Ji, Zhuangzhuang Ma, Dongwen Yang, Jingli Ma, Mengyao Zhang, Xu Li, Meng Wang, Ying Li, Xu Chen, Di Wu, Xinjian Li, Chongxin Shan, Zhifeng Shi

**Affiliations:** 1 Key Laboratory of Materials Physics of Ministry of Education, School of Physics, Zhengzhou University, Zhengzhou, China; 2 School of Integrated Circuits and Electronics, Beijing Institute of Technology, Beijing, China

**Keywords:** Electronics, photonics and device physics, Optical materials and structures

## Abstract

The development of negative photoconductivity (NPC)-related devices is of great significance for numerous applications, such as optoelectronic detection, neuromorphic computing, and optoelectronic synapses. Here, an unusual but interesting NPC phenomenon in the novel cesium cobalt chlorine (Cs_2_CoCl_4_) single crystal-based optoelectronic devices is reported, which simultaneously possess volatile resistive switching (RS) memory behavior. Joint experiment−theory characterizations reveal that the NPC behavior is derived from the intrinsic vacancy defects of Cs_2_CoCl_4_, which could trap photogenerated charge carriers and produce an internal electric field opposite to the applied electric field. Such NPC effect enables an abnormal photodetection performance with a decrease in electrical conductivity to illumination. Also, a large specific detectivity of 2.7 × 10^12^ Jones and broadband NPC detection wavelength from 265 to 780 nm were achieved. In addition to the NPC response, the resulting devices demonstrate a volatile RS performance with a record-low electric field of 5 × 10^4 ^V m^−1^. By integrating the characteristics of electric-pulse enhancement from RS and light-pulse depression from NPC, an artificial optoelectronic synapse was successfully demonstrated, and based on the simulation of artificial neural network algorithm, the recognition application of handwritten digital images was realized. These pioneer findings are anticipated to contribute significantly to the practical advancement of metal halides in the fields of in-memory technologies and artificial intelligence.

## Abstract (web summary)

The novel Cs_2_CoCl_4_ single crystals optoelectronic devices demonstrate negative photoconductivity response and volatile resistive switching simultaneously, which enables unusual artificial optoelectronic synapse and high-precision recognition application of handwritten digital image.

## Graphical abstract

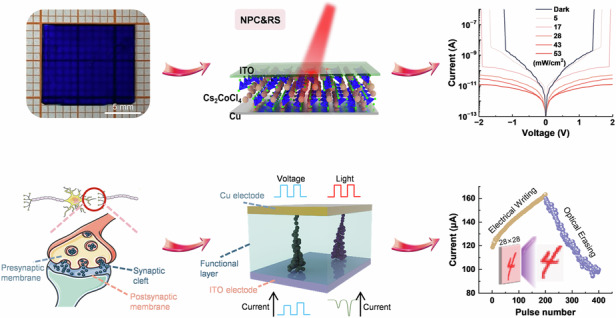

## Introduction

Today’s information technology in the fields of communication, computation, and control requires the development of optoelectronic devices that use an optical–electric conversion^1–6^. In general, semiconductors exhibit increased electrical conductivity due to the production of photoexcited carriers by light irradiation^7–11^. In some unusual situations, it is found that the conductivity of semiconductors significantly decreases under light irradiation compared to dark conditions, which is known as negative photoconductivity (NPC)^12^. Previously, this NPC phenomenon is commonly observed in low-dimensional materials^13^, for example, monolayer MoS_2_ nanosheets^14^, InAs nanowires^15,16^, SnO_2_ nanoparticles^17^, carbon nanotubes^18^, diamond^19^, and CuSe^20^. The generation of NPC in these materials is attributed to various factors, such as the introduction of acceptor deep levels, light-induced charge carrier trapping, and moisture adsorption on the surface^21–23^. With the continuous and exciting advances in this field, optoelectronic devices based on NPC characteristics have shown great potential applications in optoelectronic switches, gas detection, humidity sensors, etc., thus arousing the researchers’ strong scientific interest^24–26^.

Recently, perovskite materials have been widely used in the field of optoelectronic devices due to their high light absorption coefficients, tunable band gap, high defect tolerance, large carrier diffusion length, and low-cost solution processability^27,28^. More recently, a few perovskite materials have demonstrated NPC behavior at ambient conditions reported by different research groups. In 2019, Hague et al. observed that the photoconductivity of Bi^3+^-doped CH_3_NH_3_PbBr_3_ changed from positive to negative due to the formation of defect centers in the valence and conduction bands^29^. Subsequently, other forms of perovskites, such as CsPbBr_3_/graphene heterojunction, CsPbX_3_/ITO heterojunction, and Cs_3_Bi_2_Br_9_ thin film, were demonstrated to show NPC properties^30–32^. Although the NPC phenomenon of emerging perovskites is important and interesting from the point of view of basic photophysics and device applications, their underlying mechanism remains a mystery^33^. Equally noteworthy, in perovskite systems, complex active layers or device structures are often required to generate the NPC behavior^34–39^, such as the ion doping to introduce defect states in perovskites or the construction of multilayer heterogeneous structures. From an application perspective, the realization of considerable NPC in pristine perovskite systems without dopants or complex structures is valuable and certainly a worthwhile subject^40^.

Memristor, as a two-terminal device with the resistive switching (RS) behaviors, has the advantages of fast operation speed, low power consumption, and parallel computing since it was demonstrated a couple of decades ago^41^. Previous advances indicate that metal halide perovskites have been successfully applied in memristors and have shown excellent RS performances originating from their intrinsic ionic migration properties^42^. Benefiting from the immense advantages of perovskite materials in memristors, if combined with the unique NPC behavior, the integrated optoelectronic devices would fully coalesce the characteristics of optical electronics and memristive effects, which holds high-grade functionality and cutting-edge applications in optoelectronic systems. However, few optoelectronic devices simultaneously possess the outstanding RS performance and NPC characteristics, and researches on the related optoelectronic applications that combine the two properties remain sparse.

In this work, we adopted a novel cesium cobalt chlorine (Cs_2_CoCl_4_) single crystal (SC) with intrinsic NPC properties to construct a dual-functional Cu/Cs_2_CoCl_4_/ITO optoelectronic device, which possesses NPC response and volatile RS capability simultaneously. Joint experiment−theory characterizations provide unambiguous evidences that the intrinsic vacancy defects (*V*_Cs_ and *V*_Cl_) of Cs_2_CoCl_4_ can trap photogenerated charge carriers and generate the internal electrical fields, which oppose the external applied fields, resulting in the NPC behavior. Based on such NPC effect, an unusual photodetector was designed and fabricated, characterized by significantly lower current signals under illumination compared to the dark conditions, which also exhibits a large specific detectivity of 2.7 × 10^12^ Jones and a broadband NPC detection wavelength from 265 to 780 nm. Moreover, the fabricated devices demonstrate a volatile RS performance, reaching a high on/off ratio of 10^4^ and record-low electric field of 5 × 10^4 ^V m^−1^. By integrating NPC and RS in a single optoelectronic device, an artificial optoelectronic synapse with electric-pulse enhancement and light-pulse suppression characteristics is realized. Based on the simulation of artificial neural network algorithm, the device successfully achieves the high-precision recognition application of handwritten digital images. This innovative design offers new insights and opportunities for the application of optoelectronic devices in data processing and storage.

## Results

**Fig. 1:**
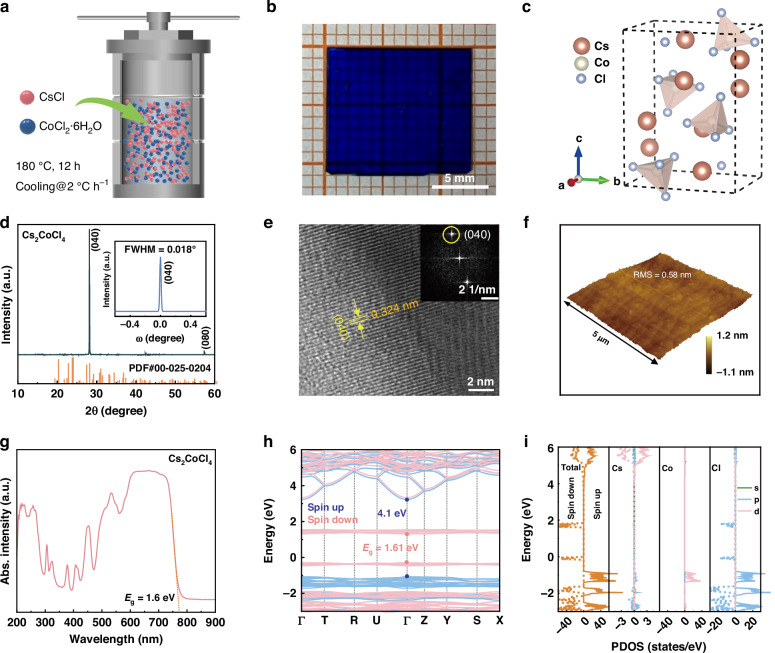
**Characterizations ofCs**_**2**_**CoCl**_**4**_**SCs.** **a** Synthetic schematic diagram of Cs_2_CoCl_4_ SCs. **b** A typical photograph of Cs_2_CoCl_4_ SC under ambient conditions. **c** Schematic crystal structure of Cs_2_CoCl_4_. **d** XRD patterns of the Cs_2_CoCl_4_ SCs. The inset shows the XRD rocking curve of the (040) diffraction peak. **e** High-resolution TEM image of the Cs_2_CoCl_4_ SCs. The inset shows the corresponding FFT patterns. **f** AFM image of Cs_2_CoCl_4_ SCs in the range of 5 μm × 5 μm. **g** Ultraviolet−visible absorption spectrum of Cs_2_CoCl_4_ SCs. **h** Calculated electronic band structure, and **i** the corresponding projected density of states of Cs_2_CoCl_4_

The Cs_2_CoCl_4_ SCs were synthesized through a meticulous slow-cooling crystallization method (see “Materials and methods” section), as illustrated in Fig. 1a. Figure 1b displays a photograph of the as-grown Cs_2_CoCl_4_ SCs, with the centimeter-scale dimensions up to 1.3 cm × 1.1 cm, being oxford-blue under daylight. Figure 1c shows the crystal structure of Cs_2_CoCl_4_, which belongs to the orthorhombic space group of *pnam*, with the unit cell parameters of *a* = 9.7710 Å, *b* = 12.9730 Å, *c* = 7.4010 Å, and *α* = *β* = *γ* = 90° (ref. ^43^), as detailed in the single-crystal-specific structure data (Tables S1 and S2). Within this structure, Co^2+^ cations are coordinated with four Cl^−^ ions, resulting in the formation of [CoCl_4_]^2^^–^ tetrahedra, and each [CoCl_4_]^2^^–^ tetrahedra are separated by Cs^+^ ions, forming a zero-dimensional crystal structure. Figure 1d depicts the typical X-ray diffraction (XRD) patterns of Cs_2_CoCl_4_ SCs, in which two intense diffraction peaks at 27.490° and 56.707° correspond to the (040) and (080) planes, respectively, verifying an oriented preferential growth of Cs_2_CoCl_4_ SCs. These highly oriented diffraction peaks are consistent with the reference standard card (PDF#00-025-0204) and no impurity diffraction peaks appear, suggesting a high phase purity of the prepared Cs_2_CoCl_4_ SCs. The inset in Fig. 1d shows the full-width at half-maximum (FWHM) of (040) diffraction peak from the high-resolution X-ray rocking curve, with a value as low as 0.018°, much lower than the previously reported CH_3_NH_3_PbBr_3_ (0.113°) and Cs_3_Bi_2_I_9_ (0.058°) SCs^44,45^, suggesting an excellent crystalline quality of the prepared Cs_2_CoCl_4_ SCs. Figure S1 presents the survey X-ray photoelectron spectroscopy (XPS) spectrum of Cs_2_CoCl_4_ SCs, and the characteristic signals of Cs, Co, and Cl were probed. Specifically, the two peaks of Co 2p can be fitted with the binding energies of 793.9 eV and 778.2 eV, which correspond to the Co 2p_1/2_ and Co 2p_3/2_, respectively. The binding energies of Cl 2p_1/2_ and Cl 2p_3/2_ lie in 197.0 eV and 195.4 eV, matching well with the previously reported chlorine-based perovskites^46^.

Figure S2 presents the scanning electron microscope (SEM) images of Cs_2_CoCl_4_ SCs, revealing a dense and uniform morphology without grain boundary. The corresponding elemental distribution map obtained from the energy-dispersive X-ray spectroscopy (EDS) analysis clearly shows that the Cs, Co, and Cl elements are evenly distributed throughout the entire detected areas. The quantitative atomic ratio (%) of Cs:Co:Cl was determined to be 2.00:1.09:4.05, which is quite consistent with the stoichiometry of Cs_2_CoCl_4_ (Fig. S3). Figure 1e presents the high-resolution TEM image of Cs_2_CoCl_4_ SCs, and a set of lattice fringes with the interplanar space of 0.324 nm was observed, corresponding to the (040) plane of orthorhombic Cs_2_CoCl_4_. The corresponding fast Fourier transform (FFT) image shown in the inset of Fig. 1e confirms the single-crystal nature of the Cs_2_CoCl_4_ SCs^47^. Figure 1f shows the atomic force microscopy (AFM) image of Cs_2_CoCl_4_ SCs, which exhibits an exceptionally low root-mean-square roughness of only 0.58 nm within a 25 μm^2^ area, indicating a smooth and homogenous surface. Such a smooth morphology with no grain boundary of Cs_2_CoCl_4_ SCs is beneficial for the preparation of optoelectronic devices^27,48^.

The optical properties of Cs_2_CoCl_4_ SCs were investigated by ultraviolet−visible absorption spectra at room temperature. As shown in Fig. 1g, a quite sharp absorption edge at approximately 770 nm was observed. The Tauc plot depicted from the absorption spectrum is presented in Fig. S4a, deriving a band gap of 1.6 eV, along with large absorption coefficient over 10^5 ^cm^−1^ above the band gap (Fig. S4b). Moreover, the Cs_2_CoCl_4_ SCs present a creditable storage stability, confirmed by the fact that the absorption spectra and structural integrity can be maintained after storage in environmental conditions (25 °C, 45–65% humidity) for one year (Fig. S5). We further conducted the ultraviolet photoelectron spectroscopy (UPS) of Cs_2_CoCl_4_ SCs to examine their valence band maximum (VBM) and conduction band maximum (CBM) distribution. As depicted in Fig. S6, the binding energy of the secondary cutoff point (*E*_cutoff_) was measured to be 16.67 eV, and the difference between VBM and Fermi energy level (*E*_Fermi_) was found to be 0.91 eV. According to the following equations of *E*_VBM_ = *hν* – *E*_Cutoff_ + *E*_Femi_ and *E*_CBM_ = *E*_g_ + *E*_VBM_, we got the *E*_VBM_ and *E*_CBM_ of Cs_2_CoCl_4_ as 5.46 eV and 3.86 eV, respectively (Fig. S7). To understand the electronic structure of Cs_2_CoCl_4_, the electronic band structure and projected density of states (DOS) were calculated based on the density functional theory. The calculated band structure presents two band gaps (Fig. 1h), denoted as a spin-down band gap of 1.61 eV (corresponding to the absorption edge at ≈770 nm) and a spin-up band gap of 4.10 eV (corresponding to the high-energy absorption location at ≈300 nm). The corresponding DOS calculation of Cs_2_CoCl_4_ covering spin properties is shown in Fig. 1i. For spin-up and spin-down channels, the conduction band and valence band of Cs_2_CoCl_4_ mainly originate from the Co 3*d* and Cl 3*p* orbitals. The Cs cation contribution to conduction band and valence band is too little to be considered.

**Fig. 2:**
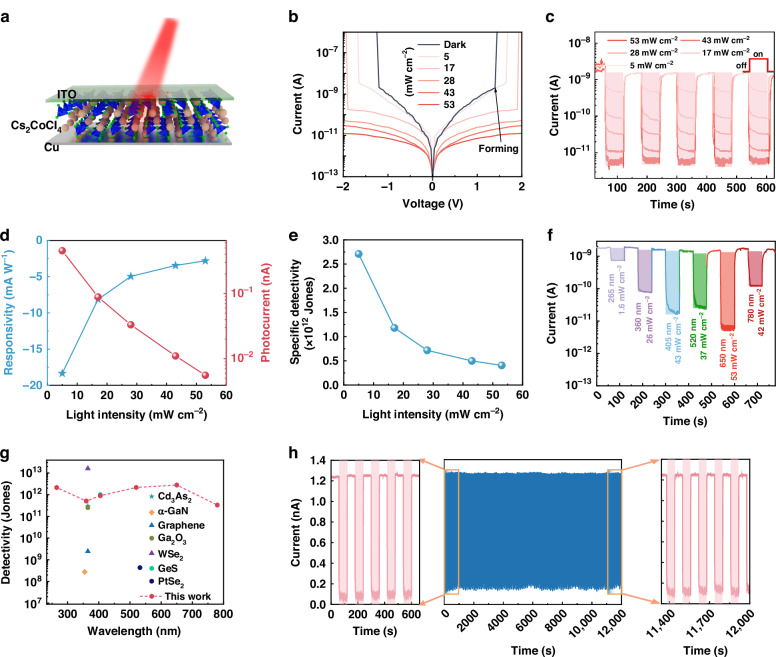
**NPC performances of the Cu/Cs**_**2**_**CoCl**_**4**_**/ITO device**. **a** Schematic diagram of the Cu/Cs_2_CoCl_4_/ITO structured photodetector. **b** *I*−*V* curves under dark and 650 nm light illumination with various light power density. **c** *I*−*t* curves of the device under different light power density at 650 nm. **d** Photocurrent and photoresponsivity of the device *versus* light intensity at 650 nm. **e** Specific detectivity of the photodetector as a function of light intensity. **f** Time-resolved photoresponse of the device under light irradiation in a broadband spectral range (265 − 780 nm). **g** Comparison of detectivity of our device with previously reported NPC response devices. **h** Stability study of the Cs_2_CoCl_4_ SCs photodetector with 300 photoresponse cycles

Further, we designed a vertically structured optoelectronic device (Fig. 2a) with a structure of Cu/Cs_2_CoCl_4_/ITO (actual device is shown in Fig. S8) to assess the photoresponse capability of Cs_2_CoCl_4_ SCs due to its good absorption properties. Figure 2b illustrates the current−voltage (*I* − *V*) characteristics of photodetector under dark and illumination with different light power densities (650 nm laser). Note that the photocurrent is lower than the dark current, which suggests a NPC response of the fabricated device^12^, accompanied by a sudden change in resistance at different voltages, which will be further discussed later. Figure 2c presents the time-dependent current (*I*−*t*) characteristics (the period is 2 minutes) of the device under illumination with different light power densities. Notably, with the increase of incident light intensity, the photocurrent exhibits a diminishing trend, characterized by a significant NPC phenomenon. Significantly, at a light power density of 53 mW cm^−2^, the device yields a switch ratio of 246. Moreover, we investigated the photocurrent change of the device under 20 s illumination (650 nm, 20 mW cm^−2^). As shown in Fig. S9, during the 20 s illumination process, the photocurrent continues to decrease, and then exhibits a slow decay over time when the light is turned off. These phenomena correspond to the biological synapse behaviors, indicating the possibility of application of such device in the field of artificial synapses.

To quantitatively evaluate the NPC response capability of the proposed device, the light responsivity (*R*) is calculated by the following formula:

1 $$R=\frac{{I}_{L}-{I}_{D}}{PS}$$

where *I*_L_ represents the photocurrent, *I*_D_ represents the dark current, *P* is the light intensity, and *S* represents the active region. As shown in Fig. 2d, the responsivity decreases gradually as the incident light intensity increases, and when the light intensity is 5 mW cm^−2^, the peak responsivity reaches –18.3 mA W^−1^. Another quantity characterizing the photodetector performance is the specific detectivity (D^*^), and the relationship is${D}^{\ast }=\frac{{A}^{1/2}R}{(2e{I}_{D})}$, where *A* is illuminated area, *R* is light responsivity, and *e* is the fundamental charge. As shown in Fig. 2e, the maximum value of D^*^ is calculated to be 2.7 × 10^12^ Jones. Figure 2f illustrates the *I* − *t* characteristics under various illumination conditions with different detection wavelengths. Note that the photodetector exhibits a broadband NPC response range from 265 nm to 780 nm. Figures S10–14 specifically show the *I* − *t* curves of the device at various detection wavelengths of 265 nm, 360 nm, 405 nm, 520 nm, and 780 nm, respectively. All the results indicate that the NPC characteristics of the device are independent of the detection wavelength, suggesting that such NPC effect is closely related to the intrinsic Cs_2_CoCl_4_ SCs active layer. Note that our devices deliver large specific detectivities in the detection range of 265 − 780 nm, which is higher than that of other traditional semiconductor-based devices with NPC (Fig. 2g and Table S3). Figure 2h exhibits the on/off cycle stability of device without encapsulation, showing that the NPC response signal can be well maintained after 300 consecutive cycles, indicating a good stability and repeatability of Cs_2_CoCl_4_ SCs-based photodetector.

**Fig. 3:**
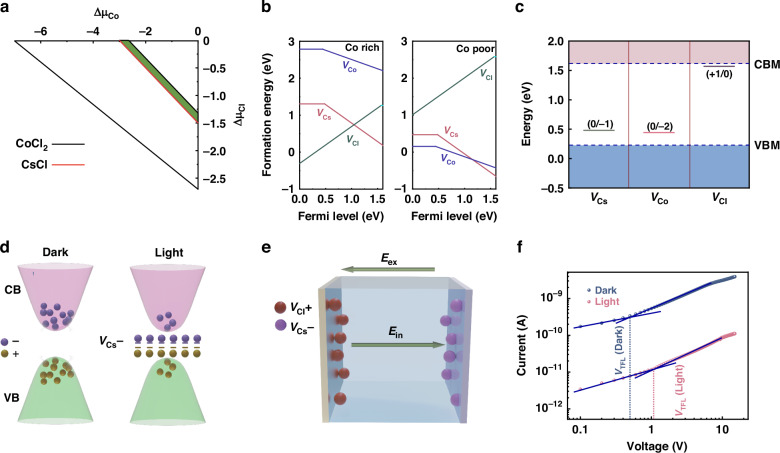
**The NPC mechanisms of Cs**_**2**_**CoCl**_**4**_. **a** Calculated phase stability diagram of Cs_2_CoCl_4_ against Δ*μ*_Co_ and Δ*μ*_Cl_. **b** Formation energies of the intrinsic vacancy defects as a function of Fermi level. **c** Transition energy levels of intrinsic vacancy defects in Cs_2_CoCl_4_. **d** Variation of the charge carriers in the conduction band and valence band under dark and illumination conditions. **e** Schematic representation of the NPC mechanism caused by the vacancies and charged carriers. **f** *I*−*V* curves of the device under dark and illumination conditions in a double-logarithmic scale

In general, in perovskite systems, the intrinsic defect states can trap the photogenerated charge carriers and generate an internal electric field that is the opposite of the externally applied electric field, thus resulting in the NPC phenomenon^12^. To gain a deeper understanding of the mechanism of NPC in Cs_2_CoCl_4_ SCs, we conducted the first-principles calculations to study the intrinsic defects of Cs_2_CoCl_4_. Firstly, under thermodynamic equilibrium growth conditions, the formation of Cs_2_CoCl_4_ could satisfy the following constraints:

2 $$2\Delta {\mu }_{{\rm{Cs}}}+\Delta {\mu }_{{\rm{Co}}}+4\Delta {\mu }_{{\rm{Cl}}}=\Delta {H}_{{{\rm{Cs}}}_{2}{{\rm{CoCl}}}_{4}}$$

3 $$\Delta {\mu }_{i}\le 0(i={\rm{Cs}},{\rm{Co}},{\rm{Cl}})$$

4 $$\Delta {\mu }_{{\rm{Cs}}}+\Delta {\mu }_{{\rm{Cs1}}}\le \Delta {H}_{{\rm{CsC1}}}$$

5 $$\Delta {\mu }_{{\rm{Co}}}+2\Delta {\mu }_{{\rm{C1}}}\le \Delta {H}_{{{\rm{CoC1}}}_{2}}$$

where $\Delta {\mu }_{{\rm{i}}}={\mu }_{{\rm{i}}}-{\mu }_{{\rm{i}}}^{{\rm{bulk}}}$ is the deviation of the chemical potential of atomic species, Δ*H* is formation enthalpy. Equation (2) is the thermodynamic equilibrium, Eq. (3) is used to prevent atomic species from precipitating into elemental phases, and Eqs. (4) and (5) are used to avoid the formation of any secondary competing phase, such as CsCl and CoCl_2_. As shown in Fig. 3a, the chemical potential window against Δ*μ*_Co_ and Δ*μ*_Cl_ satisfying Eqs. (2)–(5) is depicted as the green area. Herein, three vacancy defects (*V*_Cs_, *V*_Co_, *V*_Cl_) were considered in Cs_2_CoCl_4_, as vacancy defects tend to be generated by common ion migration^33^. Since the formation energies of point defects depend on the chemical potential of the constituent elements, two representative points, Co-rich and Co-poor, were selected to calculate the formation energies. Figure 3b illustrates the formation energies of three vacancy defects in Cs_2_CoCl_4_ as a function of the Fermi level, in which the *V*_Cl_ and *V*_Cs_ defects possess relatively low formation energies at Co rich conditions, indicating that both types of defects are easily formed. At Co poor conditions, the calculated formation energies of *V*_Cs_ and *V*_Co_ are the lowest. Due to the characteristic of Co rich in the experimentally prepared Cs_2_CoCl_4_, *V*_Cl_ and *V*_Cs_ defects are more likely to exist in Cs_2_CoCl_4_. Moreover, we calculated the energy level positions of three vacancy defects in Cs_2_CoCl_4_, as shown in Fig. 3c. One can observe that the *V*_Cs_ and *V*_Co_ in Cs_2_CoCl_4_ can create deep energy levels within the band gap, possessing the capability to trap the charge carriers. While, the *V*_Cl_ can serve as a shallow defect. Therefore, we reasonably consider that the intrinsic defects of *V*_Cl_ and *V*_Cs_ in Cs_2_CoCl_4_ SCs can be viewed as donor levels and acceptor levels, respectively, trapping charge carriers within the band gap^49,50^. Based on the above theoretical calculation results, we illustrated the NPC mechanisms in the Cs_2_CoCl_4_ SCs. Upon photoexcitation, electrons and holes are generated and combine to form excitons as a result of Coulomb interaction. These free excitons are subsequently trapped by the *V*_Cl_ and *V*_Cs_ defects within the band gap and transformed into the charged positive (*V*_Cl_^+^) and negative (*V*_Cs_^−^) defects (Fig. 3d), as the formation energy of these charged excitons is lower than that of free excitons. These defects (*V*_Cl_^+^ and *V*_Cs_^−^) can create an internal electric field within the Cs_2_CoCl_4_ SCs (Fig. 3e), in which the direction of the internal electric field is opposite to that of the external applied electric field, resulting in a reduction of current signals under illumination than the dark conditions.

The NPC mechanism in Cs_2_CoCl_4_ SCs can further be supported by examining the conduction process under illumination and dark conditions. Figure 3f shows the *I*−*V* curves of the device in the space-charge-limited current (SCLC) mode^51–54^. The trap state density (*n*_trap_) could thereby be calculated from a measure of trap-filled limited voltage (*V*_TFL_) using the equation of *n*_trap_ = 2*εε*_0_*V*_TFL_/*ed*^2,55^, where *ε* and *ε*_0_ are the relative dielectric constants of Cs_2_CoCl_4_ (*ε* ≈ 15.2, as confirmed by Fig. S15) and vacuum permittivity, respectively, *d* is the thickness of Cs_2_CoCl_4_ SCs, and *e* is the elementary electronic charge. As shown in Fig. 3f, the *n*_trap_ of Cs_2_CoCl_4_ SCs under illumination and dark conditions were calculated to be 1.8 × 10^13^ and 8.29 × 10^12 ^cm^–3^, respectively. Such NPC response could cause a decay of photocurrent in a biexponential manner (Fig. S16), involving the formation of charged defect states and the trapping and recombination processes of photogenerated carriers^56^. The fitting results show that two time constants are 1.9 s and 14.5 s, respectively, in which the smaller time constant is related to the rapid generation of trap states, and the larger time constant is associated with the delayed trapping processes^56^. These results suggest that the defects in Cs_2_CoCl_4_ SCs significantly increase under illumination, which could trap charge carriers and reduce the conductivity^56^, thus attaining the NPC response.

**Fig. 4:**
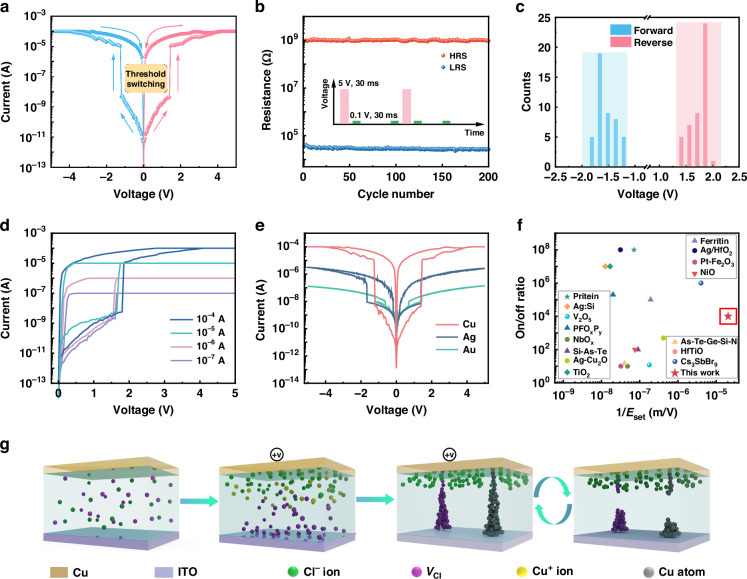
**The mechanisms and performances of resistive switching.** **a** Semilogarithmic *I*–*V* curves of the device showing threshold switching processes. **b** Endurance cycle test under pulse mode. The inset presents the applied voltage pulses for the measurement. **c** Statistics of the threshold voltages for voltage sweeps in both directions. **d** Threshold *I*–*V* curves under different compliance currents. **e** *I*−*V* curves of the memory device under different metal anodes. **f** Plots of the on/off ratio against *E*_set_ for different threshold-switching devices based on various material systems. **g** Schematic diagram of the resistance switching mechanism of the device

The fabricated Cs_2_CoCl_4_ SCs-based photodetectors not only exhibit NPC response detection ability, but also surprisingly possess the RS behavior, as mentioned earlier. Figure S17 exhibits the cross-sectional SEM images of device, showing the actual filamentation processes. Moreover, the filamentary conduction with numerous localized conductive channels could be confirmed by the conductive AFM measurements (Fig. S18). We further studied the RS behavior based on a memristor using the same device structures (Cu/Cs_2_CoCl_4_/ITO) discussed above (Fig. 2a). Figure 4a presents the electrical RS characteristic of the memristor, which is determined by applying voltage sweeps of 0 V → –5 V → 0 V → +5 V → 0 V to the top Cu electrode. When subjected to a negative bias swept from 0 to –5 V, the device rapidly transitions from a high-resistance state (HRS) to a low-resistance state (LRS), with an abrupt decrease in resistance. When the applied reverse voltage scanning process approaches 0 V, the resistance spontaneously changes from HRS to LRS. In contrast to the non-volatile bipolar resistive switching, the threshold switching in our device is naturally unipolar when the bias voltages of the opposite polarity are applied, as evidenced by the detection of symmetric hysteresis loops^57–61^. Such a device has the ability to return on its own to the HRS without the use of bias voltages with the opposite polarity. The area-dependent threshold switching is shown in Fig. S19. The constant LRS with the increasing device area reveals the localized filamentary conduction, whereas the decrease in HRS with increasing device area may be featured to the current flow through the whole area of the device, enlarging the carrier transport channels, resulting in high conductivity performance^58^. The influence of thickness on threshold switching and photocurrent properties is shown in Fig. S20. One can see that as the thickness of Cs_2_CoCl_4_ layer increases, the threshold voltage of device shows an increasing trend, along with a decrease in photocurrent. The cyclic voltage sweep measurement of the device is presented in Fig. 4b and Fig. S21, which effectively distinguishes between the HRS and LRS. Notably, the on/off ratio of the device remains consistently greater than 10^4^ throughout the entire measurement without any significant changes. The excellent reproducibility of the device is supported by the small distribution observed in the extracted threshold voltage distribution (Fig. 4c). These results indicate that the device exhibits minimal variations in its threshold voltage, highlighting their remarkable consistency and reliability^37,62,63^. The *I*−*V* readings at different compliance currents are illustrated in Fig. 4d. It is observed that the compliance current not only acts as a safeguard against device breakdown but also influences the form and diameter of the conductive filaments. At low compliance currents, the conductive filaments formed in the device are extremely delicate and tend to rupture spontaneously upon removal of the electric field. Conversely, higher compliance currents lead to the formation of thicker and more stable conductive filaments that are less prone to dissolution^49,64–66^. Moreover, the use of different top electrode materials results in various RS characteristics, as shown in Fig. 4e. The performance of devices made with Ag and Au as the top electrode are shown in Figs. S22 and Fig. S23, respectively. One can observe that the optimum RS performance is obtained by the Cu electrode because of its active nature and simplicity in oxidation-reduction processes^43^. The conduction mechanism of Au electrode-based devices is attributed to the formation of conductive filaments from Cl^–^ migration and the accumulation of defects in Cs_2_CoCl_4_ SCs, as confirmed by the temperature-dependent dark *I*−*V* measurements. As shown in Fig. S24, the increase in temperature leads to the decrease in the HRS and threshold voltage of device, which is caused by the fast Cl vacancies migration at high temperature. The EDS element distributions in line scanning confirm the filamentary conduction process dominated by Cl^–^ migration (Fig. S25). Figure 4f summarizes the on/off ratio *versus* the electric field of SET operation (*E*_set_) based on various material systems, showing that the Cs_2_CoCl_4_ SCs-based device exhibits exceptional electrical performance. To the best of our knowledge, the resulting electrical field of 5 × 10^4 ^V m^–1^ in our device is the lowest among previously documented devices (Table S4).

According to our previous theoretical results, the possible mechanism of the RS process for Cs_2_CoCl_4_ SCs-based device is illustrated in Fig. 4g. The device is initially put in the HRS by randomly dispersing the Cs_2_CoCl_4_ SCs using Cl^–^ and *V*_Cl_^+^. When the Cu electrode is subjected to positive bias voltage, Cl^–^ within the material migrates toward the Cu electrode, while the produced *V*_Cl_^+^ migrates toward the ITO electrode. Once there is enough *V*_Cl_^+^ to construct a conductive route linking the top and bottom electrodes at the ITO end, it can aggregate towards the Cu electrode through diffusion motion. Meanwhile, Cu atoms undergo an oxidation process to produce Cu^+^ ions that move in the direction of the bottom electrode. These Cu atoms continue to build up at the interface, leading to the formation of conductive filaments and changing the resistance state of the device from HRS to LRS. Because the fragile filaments are sensitive to the thermal driving force, the device can spontaneously transforms back to HRS accompanied by threshold switching starting from the origin point. Moreover, we studied the current behavior of the device under continuous voltage pulse stimulation (Fig. S26). It can be seen that the current response of the device increases nearly linearly with the increase of the applied pulses number. The incremental rise in current is attributed to the breakage of the formed filaments, requiring a specific relaxation time. This phenomenon is consistent with the synaptic plasticity in the human system, revealing the potential application of the device in artificial synapses area.

**Fig. 5:**
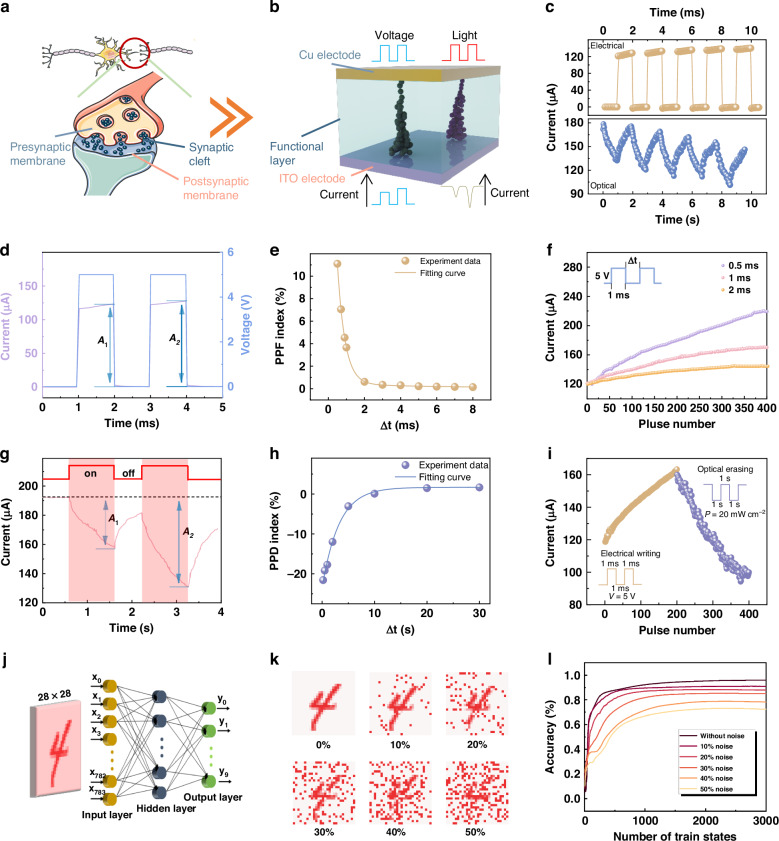
**Characterizations of electrically and optically stimulated synapses.** **a** Schematic diagram of the synaptic information transmission. **b** Schematic diagram of the optoelectronic memristor. **c** EPSC and IPSC as a function of spike number. **d** PPF behaviors for the device stimulated by two successive electrical pulses. *A*_1_ and *A*_2_ represent the current amplitude excited by the first and second electrical pulses. **e** PPF index as a function of the interval of electrical pulse pairs. **f** The changes in current of the device measured at different intervals between voltage pulses. **g** PPD behaviors for the device stimulated by two successive light pulses. *A*_1_ and *A*_2_ represent the current amplitude excited by the first and second light pulses. **h** PPD index as a function of the interval of light pulse pairs. **i** Electrical potentiation and light depression in the device. **j** Schematic of an ANN for recognizing handwritten digits with 28 × 28 pixels. **k** Handwritten digits with different noise ratios. **l** Recognition accuracy with different noise ratios

Figure 5a shows a schematic of a synapse, which is composed of presynaptic membrane, synaptic cleft, and postsynaptic membrane. The pre-synapse can release excitatory or inhibitory neurotransmitters under stimulation, and these neurotransmitters can connect with the receptors on the post-synapse, resulting in an excitatory or inhibitory postsynaptic current (EPSC/IPSC)^67^. For the Cu/Cs_2_CoCl_4_/ITO device proposed in this work, the controllable electric-pulse enhancement and NPC response with prolonged decay time enables the device to emulate both excitatory and inhibitory synaptic behaviors (Fig. 5c). Figure 5b depicts a synaptic device based on Cu/Cs_2_CoCl_4_/ITO structure, in which the conductive filament is driven by Cu ions and Cl vacancies, providing electrical and light stimulation as action potential for emulating several important synaptic functions^56^. First, paired pulse facilitation (PPF) behavior was studied, which is regarded as a typical form for short-term plasticity (STM) to recognize and decode transitory information^68^. As shown in Fig. 5d, the EPSC triggered by the second electrical pulse (*A*_2_) is larger than the first one (*A*_1_) (5 V, duration of 1 ms). As mentioned above, when the pulse interval is sufficiently short, the filament formed by the first pulse is not entirely broken. Upon applying the second pulse, the filament is reinforced, leading to increased conductance and current. The PPF index: (*A*_2_ − *A*_1_)/*A*_1_ × 100%^69,70^, was simulated by two identical electrical pulses at different time intervals (*t*) to describe the behavior of the device in the PPF test. As plotted in Fig. 5e, as *t* increased, the PPF index exhibits a declining trend, which could be fitted through the following double-exponential function^71^:

6 $$\,{\rm{PPF}}/{\rm{PPD}}={C}_{1}\exp \left(-\frac{t}{{\tau }_{1}}\right)+{C}_{2}\exp \left(-\frac{t}{{\tau }_{2}}\right)$$

The time interval between two stimulations is denoted by *t* in this equation; the initial facilitation magnitudes of the corresponding phases are indicated by *C*_1_ and *C*_2_, and the characteristic relaxation time constants for the rapid and slow decay terms are represented by *τ*_1_ and *τ*_2_, respectively. The fitted values for *τ*_1_ and *τ*_2_ under positive voltage pulses are 343 μs and 4.23 ms, respectively. This result is compatible with the values in the biological synapse^72^.

Then, we studied the current response behavior of the device at different electrical pulse intervals (Fig. 5f). The increase in current is less significant at larger pulse interval, likely because the newly formed conductive filaments at longer pulse intervals tend to break more before the arrival of the next pulse. This observation suggests that the growth of conductive filaments in the device originates from the remnants of existing conductive filaments rather than starting from zero^73^. This behavior lays the foundation for realizing long-term memory (LTM). Correspondingly, paired-pulse depression (PPD) is other forms of STM in the inhibitory synapse. As shown in Fig. 5g, the IPSC triggered by the second light pulse (*A*_2_) is significantly lower than the first one (*A*_1_). The PPD index (Fig. 5h) also shows a falling trend as the *t* increases. According to Eq. (6), the time constants of *τ*_1_ and *τ*_2_ were calculated as 10 ms and 419 ms, respectively. Figure S27 exhibits the IPSC curves stimulated by one pulse with a variable light width (2, 4, 6, 8, 10, 15, and 20 s). As expected, a stronger light pulse stimulus induced a higher IPSC value. This enables the transition from STM to LTM, which is known as the foundation of biological memory and learning.

To explore the potential of the device in the field of neuromorphic computing, the long-term depression (LTD) and long-term potentiation (LTP) behaviors of the device were analyzed^74^. As illustrated in Fig. 5i, 200 consecutive electrical pulses with a pulse width of 1 ms and a pulse interval of 2 ms (5 V) were applied to the device, followed by another 200 light pulses with a duration of 1 s and a pulse interval of 2 s (650 nm, 20 mW cm^−2^). For neuromorphic computing, the recognition accuracy is highly dependent on the linearity of the weight update trajectory^56,75^. The nonlinearities of the LTP and LTD curves are extracted from the following equations^76^:

7 $${G}_{n+1}-{G}_{n}={\alpha }_{{\rm{p}}}{{\rm{e}}}^{{-{\beta }_{p}}^{\frac{{G}_{n}-{G}_{\min }}{{G}_{\max }-{G}_{\min }}}}({\rm{for}}\,{\rm{LTP}})$$

8 $${G}_{n+1}-{G}_{n}={-}{\alpha }_{{\rm{p}}}{{\rm{e}}}^{-{{\beta }_{p}}^{\frac{{G}_{\max }-{G}_{n}}{{G}_{\max }-{G}_{\min }}}}({\rm{for}}\,{\rm{LTD}})$$

where the parameters *α* and *β* indicate the changing step sizes of the conductance and nonlinearity, respectively, and *G*_n+1_ and *G*_n_ represent the synaptic conductance of the device in the current and updated states, respectively. *G*_max_ and *G*_min_ are the measured maximum and minimum values of *G*, respectively. The nonlinearities of the LTP and LTD curves for the device were calculated as 1.472 and 2.115, respectively (Fig. S28).

Based on the extracted parameters from the conductance update characteristics of the device, a three-layer artificial neural network (ANN) with one hidden layer was simulated to simulate the handwritten digit (28 × 28 pixels) recognition task of the modified national institute of standards and technology (MNIST) database (Fig. 5j). Here, the synaptic weight of the network is defined as the difference in conductance between two equivalent synapses: W = G^+^ − G^−^. To demonstrate the fault-tolerance capability of the network, different levels of noise (0 − 50%) were applied to the database (Fig. 5k). Figure 5l presents the recognition accuracies of the network for handwritten digits with different noise ratios. The recognition accuracies of the network can reach 95.9% after the training process of 3000 states. Notably, the recognition accuracy is still higher than 70% when the noise ratio is set to 50%, demonstrating a strong fault-tolerance capability.

## Discussion

In conclusion, we successfully synthesized the novel Cs_2_CoCl_4_ SCs via a slow-cooling crystallization method, which are characterized by an intrinsic NPC behavior. Theoretical calculation and experimental analyses rationalize that the intrinsic *V*_Cl_ and *V*_Cs_ defects of Cs_2_CoCl_4_ trap photogenerated charge carriers and generate an internal electric field opposite to the applied electric field, which is responsible for the behavior of NPC. Dual-functional devices with NPC detection response and volatile RS capability were fabricated using a sandwiched Cu/Cs_2_CoCl_4_/ITO architecture. Specifically, the devices demonstrate a remarkable NPC detection ability, achieving a large specific detectivity of 2.7 × 10^12^ Jones, a high responsivity of –18.3 mA W^−1^, and a broadband NPC detection wavelength from 265 nm to 780 nm. Moreover, a volatile RS performance was demonstrated in the same device, which reached a high on/off ratio of 10^4^ and a record-low electric field of 5 × 10^4 ^V m^−1^. Based on these findings, an artificial optoelectronic synapse was demonstrated by combining NPC and RS characteristics, showcasing electrical potentiation and optical depression. Finally, based on the simulation of artificial neural network algorithm, the device successfully realizes a high-precision recognition application of handwritten digital images. These results suggest that optoelectronic devices based on Cs_2_CoCl_4_ SCs are anticipated to play a crucial role in future data processing and storage applications.

## Materials and methods

### Materials

Cesium chloride (CsCl, 99.99%), Cobalt chloride, and hexahydrate (CoCl_2_.6H_2_O, 99.99%) were purchased from Shanghai Macklin. Hydrochloric acid (HCl, analytical reagent) was purchased from Guangzhou Chemical Reagent Factory. All these chemical materials were directly used without further purification.

### Synthesis of Cs_2_CoCl_4_ single crystals

Cs_2_CoCl_4_ SCs were synthesized by a hydrothermal method. In detail, the mixture of CsCl (2 mmol, 336.72 mg) and CoCl_2_·6H_2_O (1 mmol, 237.93 mg) was put into a mortar. After thorough grinding, the mixture was transferred to a polytetrafluoroethylene (PTFE) container and then HCl of 3 mL was added. Subsequently, the container was placed in a stainless steel autoclave and transferred to a drying oven. The heating condition is 180 °C for 12 h. To obtain high-quality Cs_2_CoCl_4_ SCs, a slow cooling tactic was performed with a cooling rate of 2 °C h^−1^. The crystals were finally obtained by washing with ethanol and drying on a heating plate at 50 °C.

### Device fabrication

Using a magnetron sputtering process with an argon flow rate of 106 sccm and a power of 30 W for 40 minutes, an ITO electrode (250 nm) is deposited on one side of the Cs_2_CoCl_4_ SCs. Thermal evaporation is used to deposit a copper electrode with a thickness of 300 nm on the opposite side. Other devices, such as Ag/Cs_2_CoCl_4_/ITO and Au/Cs_2_CoCl_4_/ITO devices, are made using the same technique.

### Characterization of materials and devices

The structure of the Cs_2_CoCl_4_ SCs was examined using XRD (TD-3500, China), and crystallinity was assessed using XRD (Panalytical; X’Pert Pro). EDS and SEM (JEOL, JSM-7500F) were employed to examine the morphology and chemical makeup of Cs_2_CoCl_4_ SCs. TEM (JEOL, JEM-3010) was used to further characterize the microstructure. AFM (Bruker; Dimension icon) was used to quantify surface roughness. The UV-visible spectrophotometer (Hitachi UH4150, UV-Vis) was used to measure the absorption spectrum. Using a digital source meter (Keithley 2636B), photovoltaic testing was carried out in ambient circumstances with room temperature. The constant voltage used for measuring photodetector performance is 1.0 V. The bias voltage for measured synaptic behavior is 5.0 V. A 650 nm laser connected to SDG 1032X arbitrary waveform generator and combined with Keithley 2636B to test the optoelectronic synaptic behavior. The pulse was generated using an SDG 1032X arbitrary waveform generator. The pulse waveforms were recorded using an oscilloscope (Tektronix DPO 2012B).

### Neural network simulation

The ANN employed in this study consists of three layers: the input layer, hidden layer, and output layer. The respective number of neurons in these layers is 784, 400, and 10. The training and recognition processes were facilitated using the back-propagation (BP) algorithm. A dataset comprising 50,000 images was utilized for training, while an additional 10,000 images were employed to assess the accuracy during testing.

### Theoretical calculations

The spin-polarized first-principles calculations were conducted using density functional theory (DFT) implemented in the VASP code, employing projector augmented-wave potentials. The wave functions of the simulated systems were expanded using a plane-wave basis set with an energy cutoff of 400 eV. The lattice parameters were held constant at their experimentally measured values, while the atomic positions were optimized using a conjugate gradient algorithm until the forces in all directions were below 0.02 eV/Å. The electronic step convergence criterion was set to within 10^–5^ eV. The electronic band structure and density of states (DOS) were calculated using the Perdew-Burke-Ernzerhof (PBE) exchange-correlation functional. To account for the influence of the delocalized d-orbitals in Co atoms, the GGA + U method was employed with a U value of 1.2 eV.

The defect properties of Cs_2_CoCl_4_ were calculated using a 2 × 2 × 2 supercell and a 1 × 2 × 1 Γ-centered k-mesh. The formation energy (∆H) of vacancy defects was determined using the following equation:

9 $$\Delta {\rm{H}}=({{E}}_{{D}}\mbox{-}{{E}}_{{H}})\mbox{-}{\sum }_{i}{n}_{i}({\mu }_{i}+{\mu }_{i}^{bulk})+q({\varepsilon }_{VBM}+{\varepsilon }_{f})$$

where *E*_*D*_ and *E*_*H*_ are the total energies of the impurity-containing and the impurity-free supercells. The number of defects and their chemical potential are represented by *n*_*i*_ and *μ*_*i*_ while *Ɛ*_VBM_ and *Ɛ*_*f*_ correspond to the VBM energy and Fermi level, respectively. For image charge correction in Cs_2_CoCl_4_, a static dielectric constant of 6.46 was employed. The charge transition level *Ɛ*(*q*/*q*’) of vacancy defects was determined by identifying the Fermi level at which the formation energy is equivalent for two different charge states, *q* and *q*’*s*:

10 $$\varepsilon =\frac{\Delta {H}_{{D,q}^{\prime}}-\Delta {H}_{D,q}}{{q-q^{\prime}}}$$

## Supplementary information

Simultaneous Achieving Negative Photoconductivity Response and Volatile Resistive Switching in Cs2CoCl4 Single Crystals towards Artificial Optoelectronic Synapse

## Data Availability

The data that support the findings of this study are available from the corresponding author upon reasonable request.

## References

[1] Leydecker, T. et al. Flexible non-volatile optical memory thin-film transistor device with over 256 distinct levels based on an organic bicomponent blend. *Nat. Nanotechnol.* **11**, 769–775 (2016). 27323302 10.1038/nnano.2016.87

[2] Yu, D. S. et al. Author correction: scalable synthesis of hierarchically structured carbon nanotube–graphene fibres for capacitive energy storage. *Nat. Nanotechnol.* **15**, 811 (2020). 32606390 10.1038/s41565-020-0718-1

[3] Nath, S. K. et al. Optically tunable electrical oscillations in oxide-based memristors for neuromorphic computing. *Adv. Mater.* **36**, 2400904 (2024). 10.1002/adma.202400904 38516720

[4] Dong, H. et al. Metal Halide Perovskite for next-generation optoelectronics: progresses and prospects. *eLight* **3**, 3 (2023).

[5] Faber, H. & Anthopoulos, T. D. Adding a new layer to ‘more than Moore’. *Nat. Electron.* **2**, 497–498 (2019).

[6] Luo, Y. M. et al. Miniature viscometer incorporating GaN optical devices with an ultrawide measurement range. *Light. Adv. Manuf.* **4**, 2 (2023).

[7] Lee, S.-J. et al. Lead halide perovskite sensitized WSe_2_ photodiodes with ultrahigh open circuit voltages. *eLight* **3**, 8 (2023).

[8] Van Gardingen-Cromwijk, T. et al. Non-isoplanatic lens aberration correction in dark-field digital holographic microscopy for semiconductor metrology. *Light. Adv. Manuf.* **4**, 36 (2023). 10.1364/OE.476157 36606976

[9] Ma, Z. Z. et al. High color-rendering index and stable white light-emitting diodes by assembling two broadband emissive self-trapped excitons. *Adv. Mater.* **33**, 2001367 (2021). 10.1002/adma.202001367 33225543

[10] Nath, S. K. et al. Schottky-barrier-induced asymmetry in the negative-differential-resistance response of Nb/NbO_*x*_/Pt cross-point devices. *Phys. Rev. Appl.* **13**, 064024 (2020).

[11] Huang, P. et al. Nonlocal interaction enhanced biexciton emission in large CsPbBr_3_ nanocrystals. *eLight* **3**, 10 (2023).

[12] Tailor, N. K. et al. Negative photoconductivity: bizarre physics in semiconductors. *ACS Mater. Lett.* **4**, 2298–2320 (2022).

[13] Cui, B. Y. et al. Negative photoconductivity in low-dimensional materials. *Chin. Phys. B* **30**, 028507 (2021).

[14] Xiao, X. L. et al. Negative photoconductivity observed in polycrystalline monolayer molybdenum disulfide prepared by chemical vapor deposition. *Appl. Phys. A* **125**, 765 (2019).

[15] Shen, L. F. et al. Enhanced negative photoconductivity in InAs nanowire phototransistors surface-modified with molecular monolayers. *Adv. Mater. Interfaces* **5**, 1701104 (2018).

[16] Yang, Y. M. et al. Hot carrier trapping induced negative photoconductance in InAs nanowires toward novel nonvolatile memory. *Nano Lett.* **15**, 5875–5882 (2015). 26226506 10.1021/acs.nanolett.5b01962

[17] Ding, L. W. et al. Graphene-skeleton heat-coordinated and nanoamorphous-surface-state controlled pseudo-negative-photoconductivity of tiny SnO_2_ nanoparticles. *Adv. Mater.* **27**, 3525–3532 (2015). 25953332 10.1002/adma.201500804

[18] Zhu, J. L. et al. Negative and positive photoconductivity modulated by light wavelengths in carbon nanotube film. *Appl. Phys. Lett.* **101**, 123117 (2012).

[19] Qin, J. X. et al. Humidity sensors realized via negative photoconductivity effect in nanodiamonds. *J. Phys. Chem. Lett.* **12**, 4079–4084 (2021). 33881881 10.1021/acs.jpclett.1c01011

[20] Singh, S. C. et al. Photothermal and joule-heating-induced negative-photoconductivity-based ultraresponsive and near-zero-biased copper selenide photodetectors. *ACS Appl. Electron. Mater.* **1**, 1169–1178 (2019). 31367704 10.1021/acsaelm.9b00174 PMC6657288

[21] Jin, H. N. et al. Positive and negative photoconductivity characteristics in CsPbBr_3_/graphene heterojunction. *Nanotechnology* **32**, 085202 (2021). 33157541 10.1088/1361-6528/abc850

[22] Lui, C. H. et al. Trion-induced negative photoconductivity in monolayer MoS_2_. *Phys. Rev. Lett.* **113**, 166801 (2014). 25361273 10.1103/PhysRevLett.113.166801

[23] Wu, J. Y. et al. Broadband MoS_2_ field-effect phototransistors: ultrasensitive visible-light photoresponse and negative infrared photoresponse. *Adv. Mater.* **30**, 1705880 (2018). 10.1002/adma.201705880 29315924

[24] Ma, Z. Z. et al. Recent advances and opportunities of eco-friendly ternary copper halides: a new superstar in optoelectronic applications. *Adv. Mater.* **35**, 2300731 (2023). 10.1002/adma.202300731 36854310

[25] Miao, J. S. et al. Photothermal effect induced negative photoconductivity and high responsivity in flexible black phosphorus transistors. *ACS Nano* **11**, 6048–6056 (2017). 28493668 10.1021/acsnano.7b01999

[26] Biswas, C. et al. Negative and positive persistent photoconductance in graphene. *Nano Lett.* **11**, 4682–4687 (2011). 21972980 10.1021/nl202266h

[27] Zhang, Y. X., Liu, Y. C. & Liu, S. Z. Composition engineering of perovskite single crystals for high-performance optoelectronics. *Adv. Funct. Mater.* **33**, 2210335 (2023).

[28] Li, H. Y. et al. Recent progress on synthesis, intrinsic properties and optoelectronic applications of perovskite single crystals. *Adv. Funct. Mater.* **33**, 2214339 (2023).

[29] Haque, M. A. et al. Transition from positive to negative photoconductance in doped hybrid perovskite semiconductors. *Adv. Opt. Mater.* **7**, 1900865 (2019).

[30] Yuan, Y. et al. Negative photoconductivity in Cs_4_PbBr_6_ single crystal. *Phys. Chem. Chem. Phys.* **22**, 14276–14283 (2020). 32555919 10.1039/d0cp02004d

[31] Tailor, N. K. et al. Dark self-healing-mediated negative photoconductivity of a lead-free Cs_3_Bi_2_Cl_9_ perovskite single crystal. *J. Phys. Chem. Lett.* **12**, 2286–2292 (2021). 33646788 10.1021/acs.jpclett.1c00057

[32] Tailor, N. K. et al. Observation of negative photoconductivity in lead-free Cs_3_Bi_2_Br_9_ perovskite single crystal. *ACS Photon.* **8**, 2473–2480 (2021).

[33] Zhang, T., Hu, C. & Yang, S. H. Ion migration: a “double-edged sword” for halide-perovskite-based electronic devices. *Small Methods* **4**, 1900552 (2020).

[34] Cheng, X. F. et al. Environmentally robust memristor enabled by lead-free double perovskite for high-performance information storage. *Small* **15**, 1905731 (2019). 10.1002/smll.201905731 31668013

[35] Tian, H. et al. Extremely low operating current resistive memory based on exfoliated 2D perovskite single crystals for neuromorphic computing. *ACS Nano* **11**, 12247–12256 (2017). 29200259 10.1021/acsnano.7b05726

[36] Liu, X. M. et al. Flexible transparent high-efficiency photoelectric perovskite resistive switching memory. *Adv. Funct. Mater.* **32**, 2202951 (2022).

[37] Mao, J. Y. et al. Lead-free monocrystalline perovskite resistive switching device for temporal information processing. *Nano Energy* **71**, 104616 (2020).

[38] Choi, J. et al. Organolead halide perovskites for low operating voltage multilevel resistive switching. *Adv. Mater.* **28**, 6562–6567 (2016). 27192161 10.1002/adma.201600859

[39] Hu, Y. Q. et al. Ultrathin Cs_3_Bi_2_I_9_ nanosheets as an electronic memory material for flexible memristors. *Adv. Mater. Interfaces* **4**, 1700131 (2017).

[40] Cao, F. et al. A dual-functional perovskite-based photodetector and memristor for visual memory. *Adv. Mater.* **35**, 2304550 (2023). 10.1002/adma.202304550 37467009

[41] Strukov, D. B. et al. The missing memristor was found. *Nature* **453**, 80–83 (2008). 18451858 10.1038/nature06932

[42] Chen, Y. C. et al. High temperature resistant solar-blind ultraviolet photosensor for neuromorphic computing and cryptography. *Adv. Funct. Mater.* **34**, 2315383 (2024).

[43] Lim, A. R. & Shin, H. W. Nuclear magnetic resonance study of the crystallographically inequivalent Cs(I) and Cs(II) in a Cs_2_CoCl_4_ single crystal. *J. Phys. Soc. Jpn* **72**, 718–722 (2003).

[44] Liu, Y. C. et al. A 1300 mm^2^ ultrahigh-performance digital imaging assembly using high-quality perovskite single crystals. *Adv. Mater.* **30**, 1707314 (2018). 10.1002/adma.201707314 29845652

[45] Zhang, Y. X. et al. Nucleation-controlled growth of superior lead-free perovskite Cs_3_Bi_2_I_9_ single-crystals for high-performance X-ray detection. *Nat. Commun.* **11**, 2304 (2020). 32385231 10.1038/s41467-020-16034-w PMC7210296

[46] Yang, R. T. et al. High-Efficiency and stable long-persistent luminescence from undoped cesium cadmium chlorine crystals induced by intrinsic point defects. *Adv. Sci.* **10**, 2207331 (2023). 10.1002/advs.202207331 PMC10214269 36825674

[47] Zhang, F. et al. Stable zero-dimensional cesium indium bromide hollow nanocrystals emitting blue light from self-trapped excitons. *Nano Today* **38**, 101153 (2021).

[48] Zhang, M. Y. et al. High-quality CsAg_2_I_3_ microwires grown by spatial confinement method for self-powered UV photodetectors. *Adv. Opt. Mater.* **11**, 2203054 (2023).

[49] Ci, P. H. et al. Chemical trends of deep levels in van der Waals semiconductors. *Nat. Commun.* **11**, 5373 (2020). 33097722 10.1038/s41467-020-19247-1 PMC7584584

[50] Bao, C. X. & Gao, F. Physics of defects in metal halide perovskites. *Rep. Prog. Phys.* **85**, 096501 (2022). 10.1088/1361-6633/ac7c7a 35763940

[51] Ma, Z. Z. et al. Carbazole-containing polymer-assisted trap passivation and hole-injection promotion for efficient and stable CsCu_2_I_3_-based yellow LEDs. *Adv. Sci.* **9**, 2202408 (2022). 10.1002/advs.202202408 PMC9507358 35780486

[52] Deng, Y. H., Yang, Z. Q. & Ma, R. M. Growth of centimeter-scale perovskite single-crystalline thin film via surface engineering. *Nano Converg.* **7**, 25 (2020). 32691332 10.1186/s40580-020-00236-5 PMC7371768

[53] Ma, J. L. et al. Kinetically regulated growth of Cs_3_Cu_2_I_5_ single-crystalline thin films for highly responsive and stable deep-ultraviolet photodetectors. *Nano Today* **52**, 101970 (2023).

[54] Chen, Y. et al. Overcoming the anisotropic growth limitations of free-standing single-crystal halide perovskite films. *Angew. Chem. Int. Ed.* **60**, 2629–2636 (2021). 10.1002/anie.202011853 33047467

[55] Liu, Y. C. et al. Two-inch-sized perovskite CH_3_NH_3_PbX_3_ (X = Cl, Br, I) crystals: growth and characterization. *Adv. Mater.* **27**, 5176–5183 (2015). 26247401 10.1002/adma.201502597

[56] Paramanik, S. & Pal, A. J. Combining negative photoconductivity and resistive switching towards in-memory logic operations. *Nanoscale* **15**, 5001–5010 (2023). 36786743 10.1039/d3nr00278k

[57] Nath, S. K. et al. Harnessing metal/oxide interlayer to engineer the memristive response and oscillation dynamics of two-terminal memristors. *Adv. Funct. Mater.* **33**, 2306428 (2023).

[58] Li, S. et al. Anatomy of filamentary threshold switching in amorphous niobium oxide. *Nanotechnology* **29**, 375705 (2018). 29939155 10.1088/1361-6528/aacee4

[59] Yan, Y. Q. et al. Multicolor vision perception of flexible optoelectronic synapse with high sensitivity for skin sunburn warning. *Mater. Horiz.* **11**, 1934–1943 (2024). 38345761 10.1039/d3mh02154h

[60] Wang, Z. R. et al. Threshold switching of Ag or Cu in dielectrics: materials, mechanism, and applications. *Adv. Funct. Mater.* **28**, 1704862 (2018).

[61] Wu, Y. et al. Capping CsPbBr_3_ with ZnO to improve performance and stability of perovskite memristors. *Nano Res.* **10**, 1584–1594 (2017).

[62] Hua, Q. L. et al. A threshold switching selector based on highly ordered ag nanodots for x-point memory applications. *Adv. Sci.* **6**, 1900024 (2019). 10.1002/advs.201900024 PMC6524079 31131198

[63] Huang, F. H. et al. Controllable resistive switching in ReS_2_/WS_2_ heterostructure for nonvolatile memory and synaptic simulation. *Adv. Sci.* **10**, 2302813 (2023). 10.1002/advs.202302813 PMC10558669 37530215

[64] Paramanik, S. & Pal, A. J. Dimensionality-dependent resistive switching in 0D and 2D Cs_3_Sb_2_I_9_: energy-efficient synaptic functions with the layered-phase. *Adv. Electron. Mater.* **8**, 2200211 (2022).

[65] Hu, J. Y. et al. Lead-free CsCu_2_Br_3_ perovskite for multilevel resistive switching memory. *Appl. Phys. Lett.* **123**, 063301 (2023).

[66] Xiao, X. Y. et al. Recent advances in halide perovskite memristors: materials, structures, mechanisms, and applications. *Adv. Mater. Technol.* **5**, 1900914 (2020).

[67] Chen, H. et al. Biological function simulation in neuromorphic devices: from synapse and neuron to behavior. *Sci. Technol. Adv. Mater.* **24**, 2183712 (2023). 36926202 10.1080/14686996.2023.2183712 PMC10013381

[68] Fiorillo, C. D. & Williams, J. T. Glutamate mediates an inhibitory postsynaptic potential in dopamine neurons. *Nature* **394**, 78–82 (1998). 9665131 10.1038/27919

[69] Tang, J. et al. A reliable all-2D materials artificial synapse for high energy-efficient neuromorphic computing. *Adv. Funct. Mater.* **31**, 2011083 (2021).

[70] Hu, L. D. et al. Ultrasensitive freestanding and mechanically durable artificial synapse with attojoule power based on na-salt doped polymer for biocompatible neuromorphic interface. *Adv. Funct. Mater.* **31**, 2106015 (2021).

[71] Wang, Y. et al. Dual-modal optoelectronic synaptic devices with versatile synaptic plasticity. *Adv. Funct. Mater.* **32**, 2107973 (2022).

[72] Wang, W. X. et al. Artificial optoelectronic synapses based on TiN_*x*_O_2–*x*_/MoS_2_ heterojunction for neuromorphic computing and visual system. *Adv. Funct. Mater.* **31**, 2101201 (2021).

[73] Wang, Z. R. et al. Memristors with diffusive dynamics as synaptic emulators for neuromorphic computing. *Nat. Mater.* **16**, 101–108 (2017). 27669052 10.1038/nmat4756

[74] Cheng, Z. G. et al. Optical synaptic devices with ultra-low power consumption for neuromorphic computing. *Light Sci. Appl.* **11**, 337 (2022). 36443284 10.1038/s41377-022-01031-z PMC9705294

[75] Xiao, F. et al. Graphene/MoS_2-*x*_O_*x*_/graphene photomemristor with tunable non-volatile responsivities for neuromorphic vision processing. *Light Sci. Appl.* **12**, 39 (2023). 36750548 10.1038/s41377-023-01079-5 PMC9905593

[76] Li, Y. et al. Flexible artificial optoelectronic synapse based on lead-free metal halide nanocrystals for neuromorphic computing and color recognition. *Adv. Sci.* **9**, 2202123 (2022). 10.1002/advs.202202123 PMC9353487 35661449

