# Supplementary material for: Simultaneous achieving negative photoconductivity response and volatile resistive switching in Cs2CoCl4 single crystals towards artificial optoelectronic synapse
Source: Light Sci Appl. 2024 Dec 2;13:316. doi: 10.1038/s41377-024-01642-8 (PMC11612145; doi:10.1038/s41377-024-01642-8)
Supplement: Supplementary file 1 — Simultaneous Achieving Negative Photoconductivity Response and Volatile Resistive Switching in Cs2CoCl4 Single Crystals towards Artificial Optoelectronic Synapse [file 41377_2024_1642_MOESM1_ESM.docx]

Supplementary Information for

Simultaneous Achieving Negative Photoconductivity Response and Volatile Resistive Switching in Cs_2_CoCl_4_ Single Crystals towards Artificial Optoelectronic Synapse

*Huifang Jiang^1#^, Huifang Ji^1#^, Zhuangzhuang Ma**^1^*, Dongwen Yang^1^, Jingli Ma^1^, Mengyao Zhang^1^, Xu Li^1^, Meng Wang^1^, Ying Li^2^*, Xu Chen^1^, Di Wu^1^, Xinjian Li^1^, Chongxin Shan^1^, and Zhifeng Shi**

^1^Key Laboratory of Materials Physics of Ministry of Education, School of Physics, Zhengzhou University, Daxue Road 75, Zhengzhou 450052, China

^2^School of Integrated Circuits and Electronics, Beijing Institute of Technology, Beijing 100081, China

***Correspondence: Zhuangzhuang Ma (mazz@zzu.edu.cn) or Ying Li (liying0326@bit.edu.cn) or Zhifeng Shi (shizf@zzu.edu.cn).


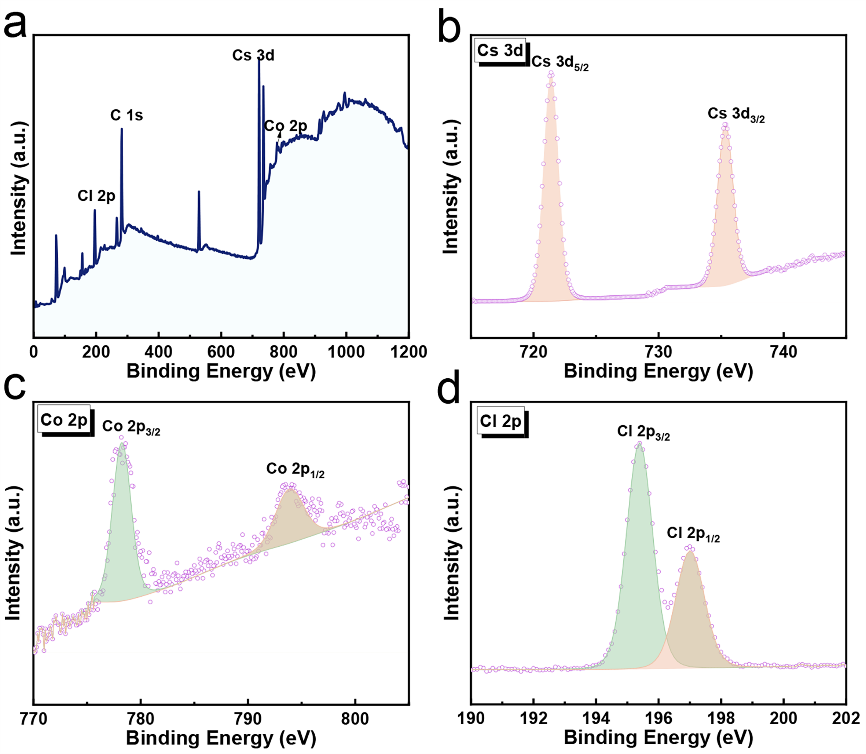


**Fig. S1** **a** The survey XPS spectrum of Cs_2_CoCl_4_ SCs, and the magnified regions of **b** Cs 3d, **c** Co 2p, and **d** Cl 2p, respectively.


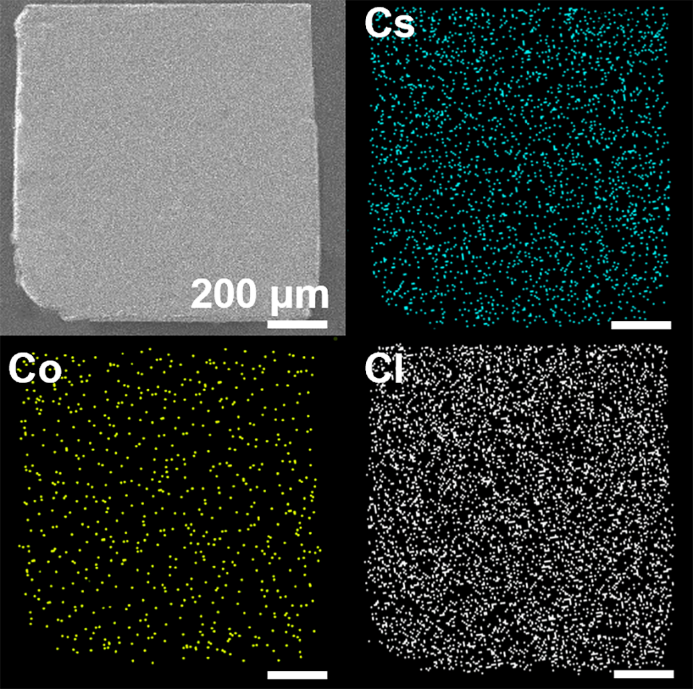


**Fig. S2** SEM image of the Cs_2_CoCl_4_ SCs and corresponding EDS elemental distribution of Cs, Co, and Cl.


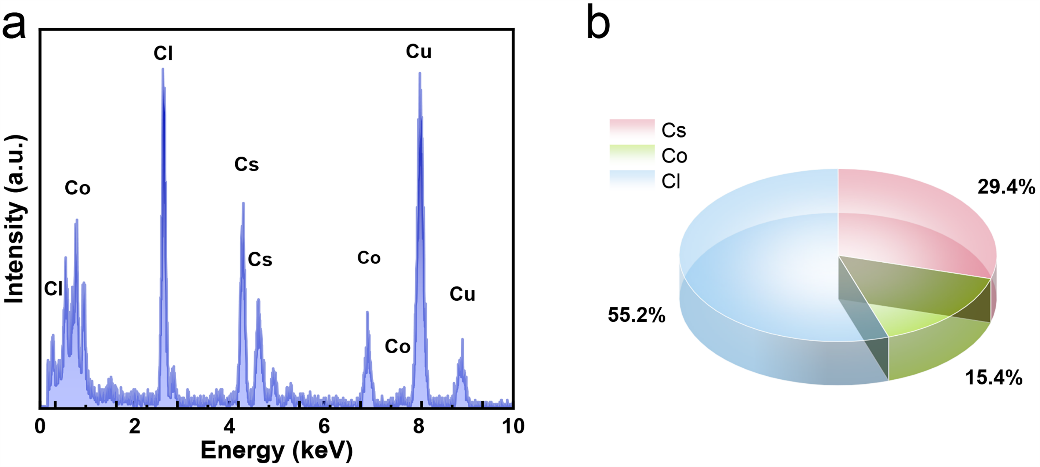


**Fig. S3 a** EDS spectrum of Cs_2_CoCl_4_. **b** The corresponding atomic ratios of the elements.


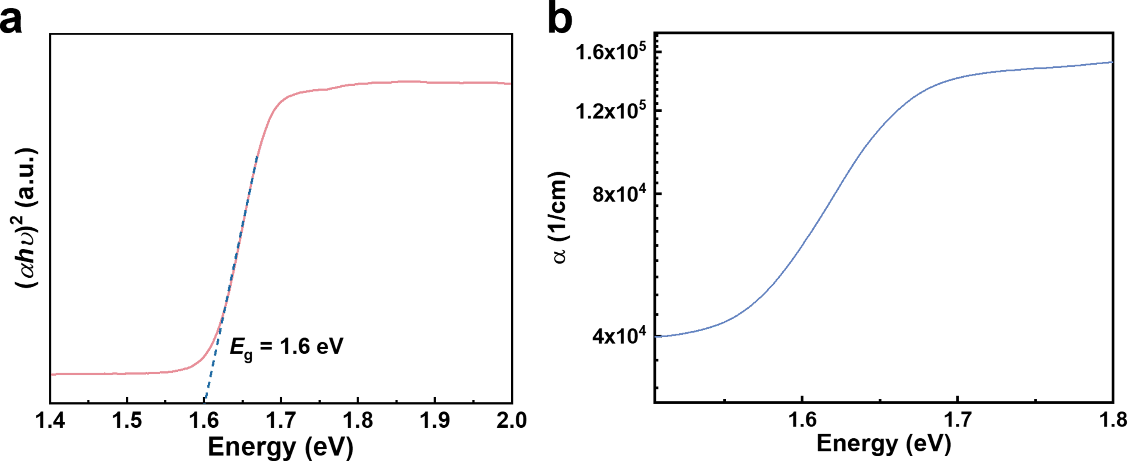


**Fig. S4** **a** Tauc plot of the absorption spectra of Cs_2_CoCl_4_ SCs. **b** Absorption coefficient of Cs_2_CoCl_4_ SCs.


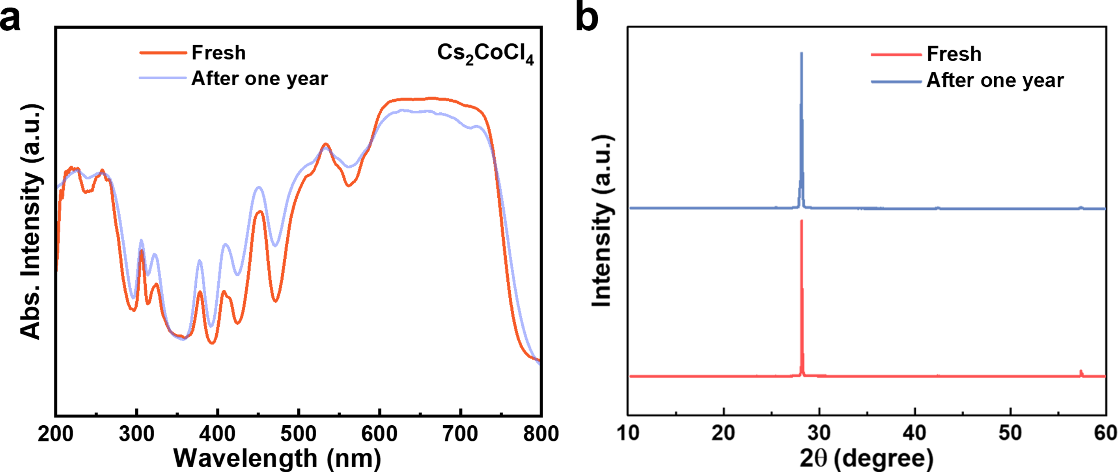


**Fig. S5** Comparison of the absorption spectra (**a**) and XRD patterns (**b**) of Cs_2_CoCl_4_ SCs before and after storage for one year in air ambient.


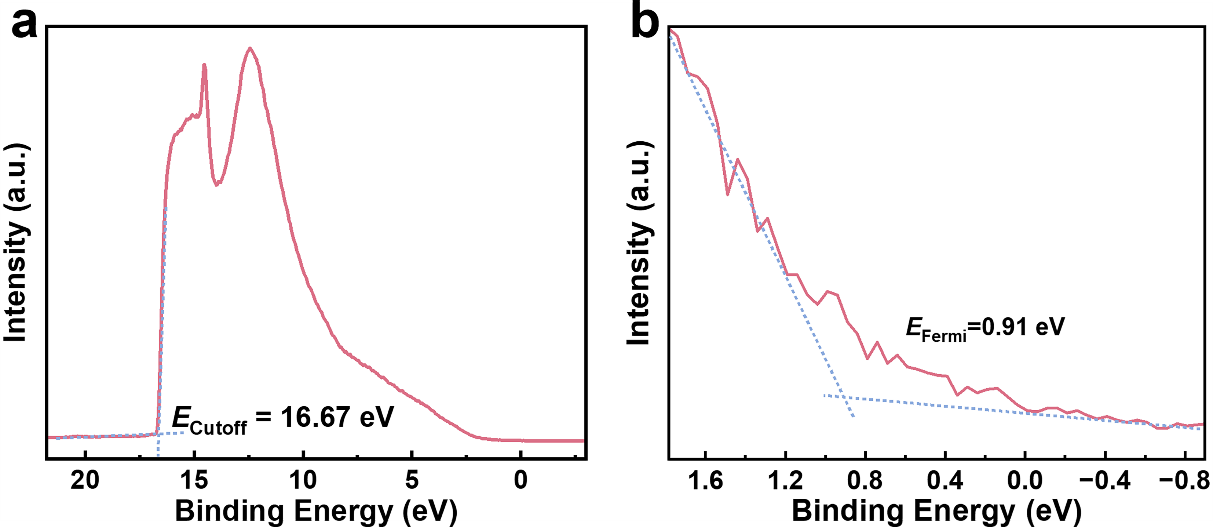


**Fig. S6** **a** UPS spectrum of the Cs_2_CoCl_4_ SCs. **b** Linear extrapolation in the low-binding-energy region.


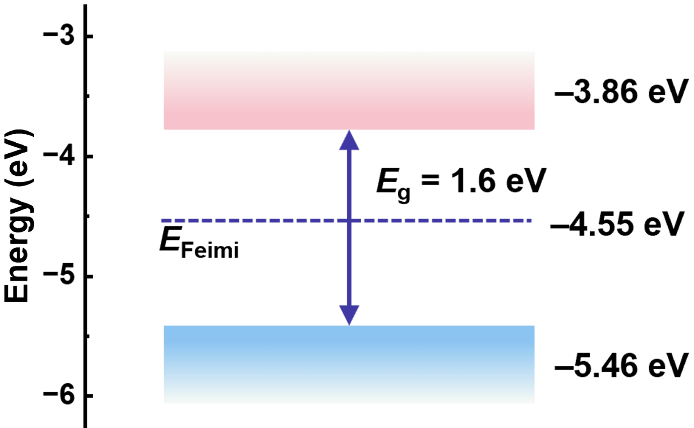


**Fig. S7** Energy band diagram of the Cs_2_CoCl_4_ SCs calculated from the Tauc plot and UPS results.


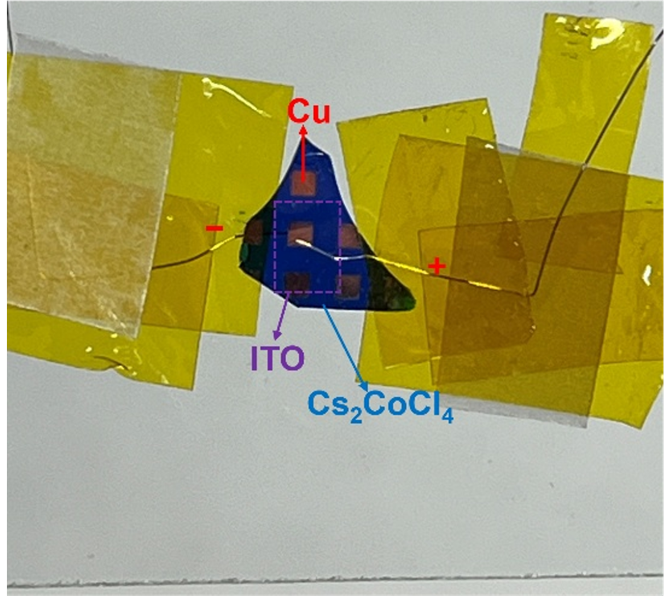


**Fig. S8** Photograph of the actual device with a structure of Cu/Cs_2_CoCl_4_/ITO.


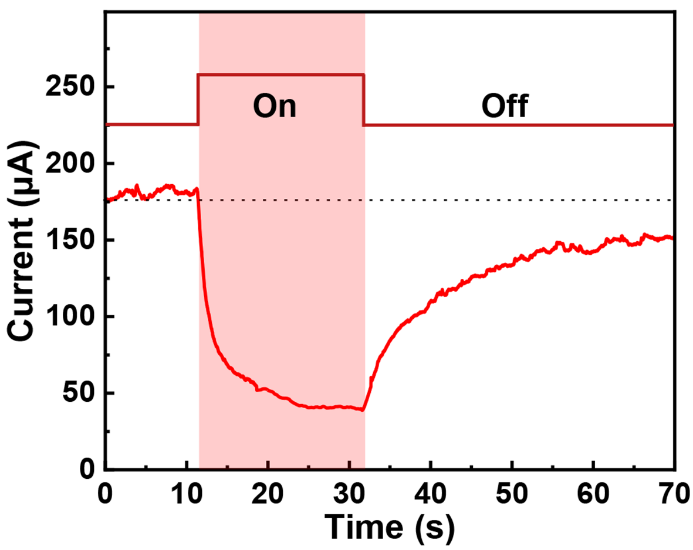


**Fig. S9** Changes in device current recorded under the illumination of red light (650 nm, 20 mW cm^−2^) at a bias of 5 V.


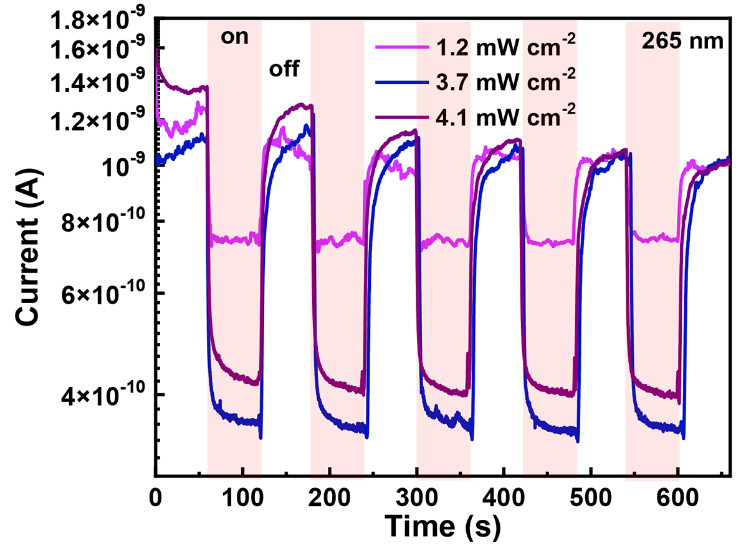


**Fig. S10** The *I*−*t* characteristics of Cu/Cs_2_CoCl_4_/ITO devices under 265 nm laser illumination.


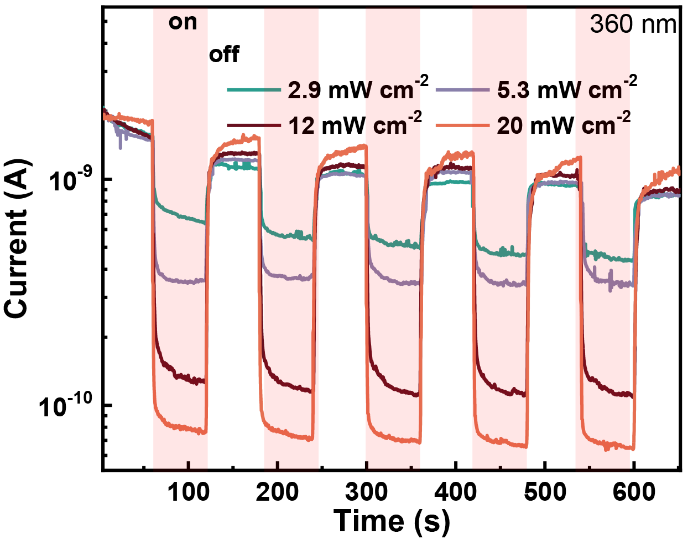


**Fig. S11** The *I*−*t* characteristics of Cu/Cs_2_CoCl_4_/ITO devices under 360 nm laser illumination.


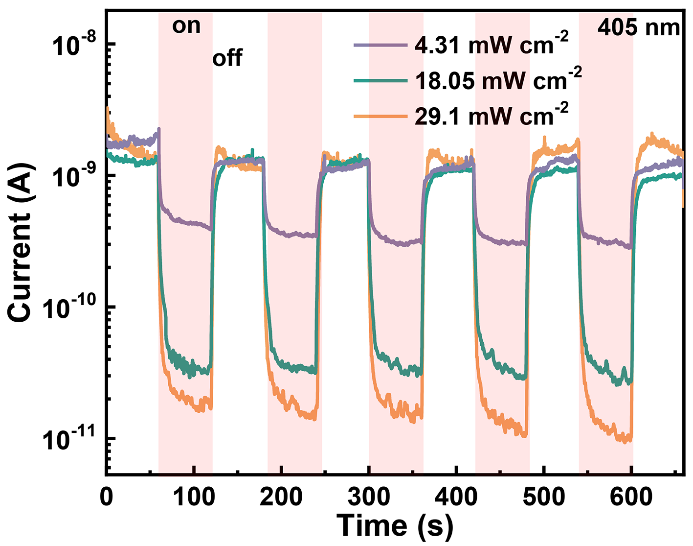


**Fig. S12** The *I*−*t* characteristics of Cu/Cs_2_CoCl_4_/ITO devices under 405 nm laser illumination.


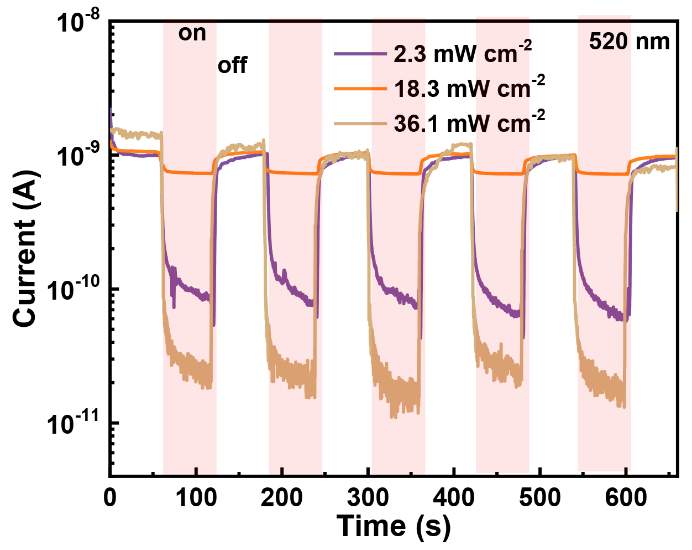


**Fig. S13** The *I*−*t* characteristics of Cu/Cs_2_CoCl_4_/ITO devices under 520 nm laser illumination.


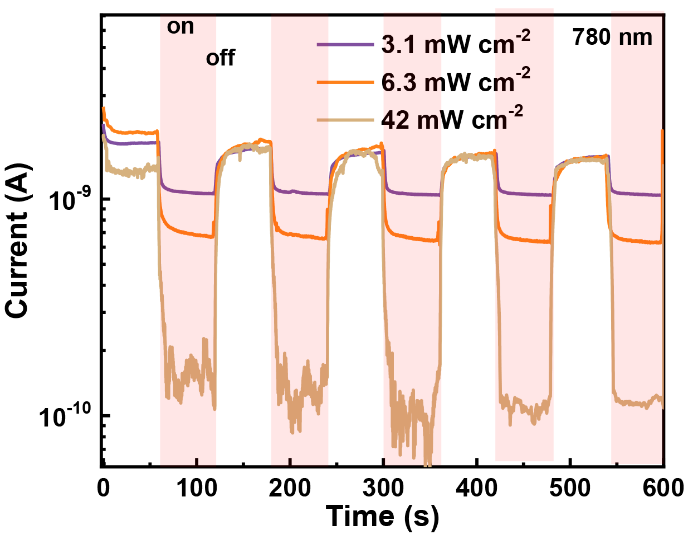


**Fig. S14** The *I*−*t* characteristics of Cu/Cs_2_CoCl_4_/ITO devices under 780 nm laser illumination.


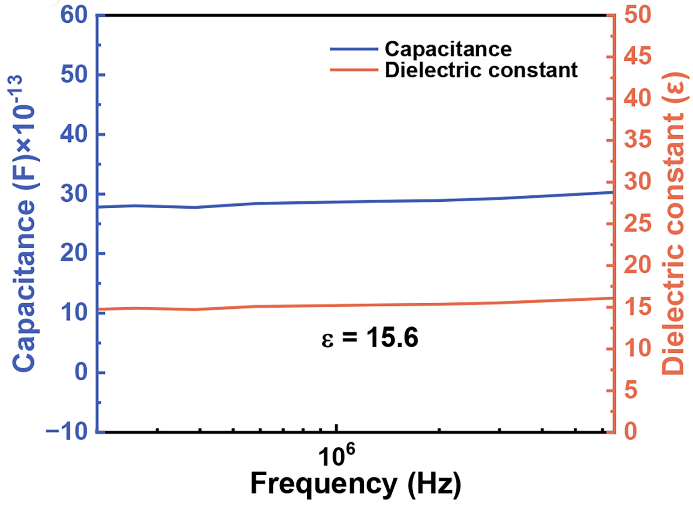


**Fig. S15** Frequency-dependent capacitance and its corresponding dielectric constant curves.


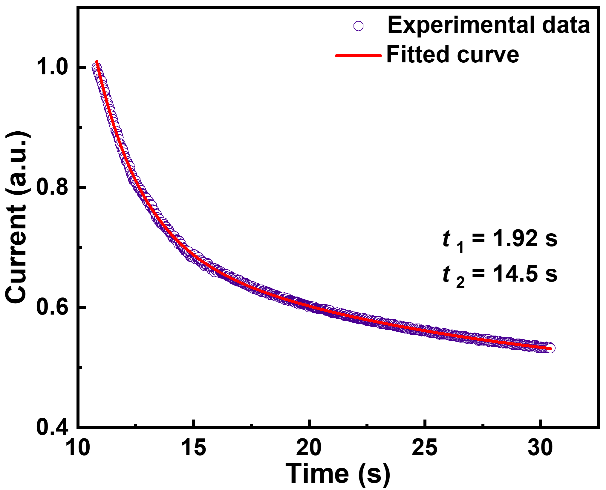


**Fig. S16** Decay characteristics of the photocurrent of device. The photocurrent decay curves can be fitted using a biexponential decay model ().


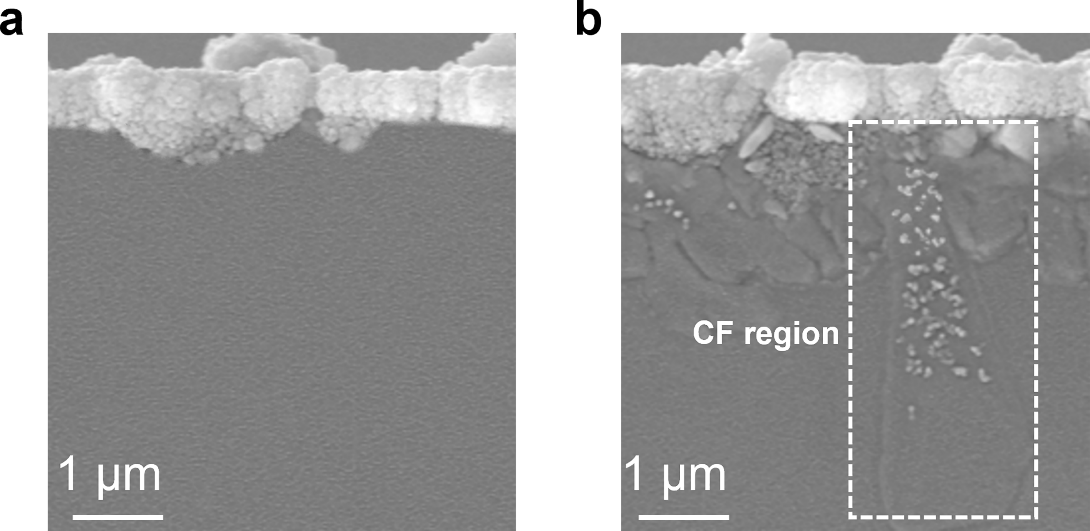


**Fig. S17** Cross-sectional SEM images of device before (**a**) and after operation (**b**) with 5.0 V bias applied.


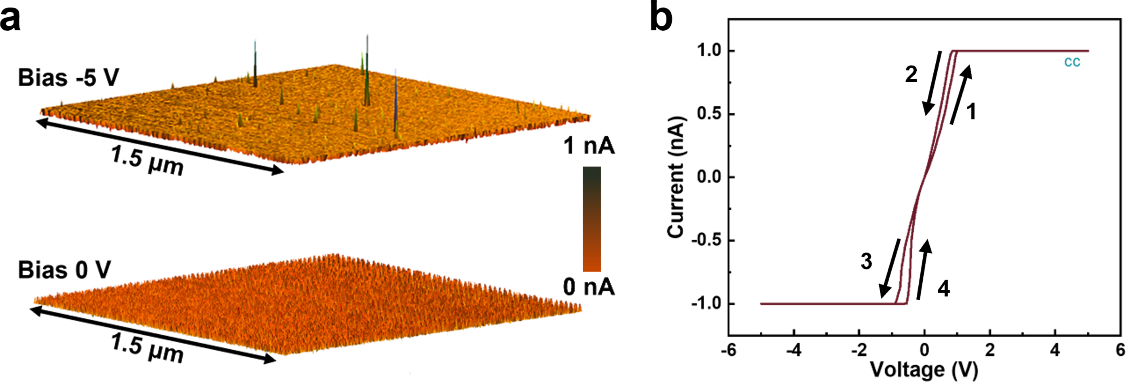


**Fig. S18** Conductive AFM measurements of device with 5.0 V bias applied.


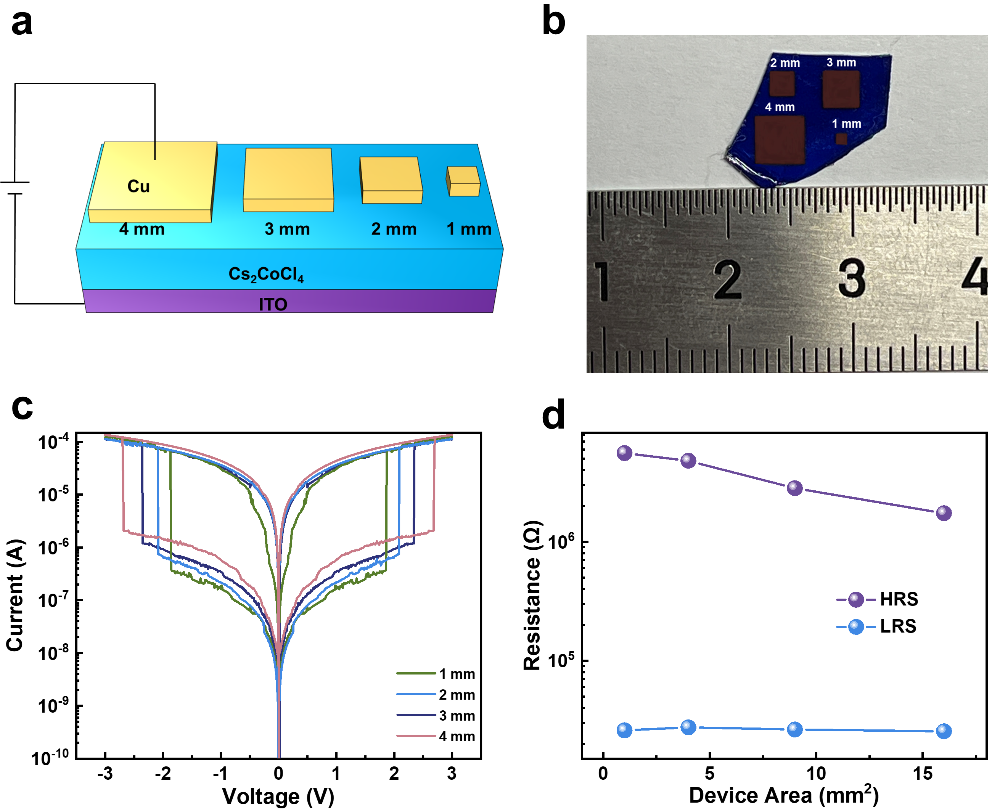


**Fig. S19** **a** Area-dependent device structure diagram. **b** Actual optical images of the Cu/Cs_2_CoCl_4_/ITO devices with electrode areas of 1 × 1 mm, 2 × 2 mm, 3 × 3 mm, and 4 × 4 mm. **c** *I−V* curves of the memristors with different electrode areas. **d** Resistance of the LRS and HRS of the device at different electrode areas.


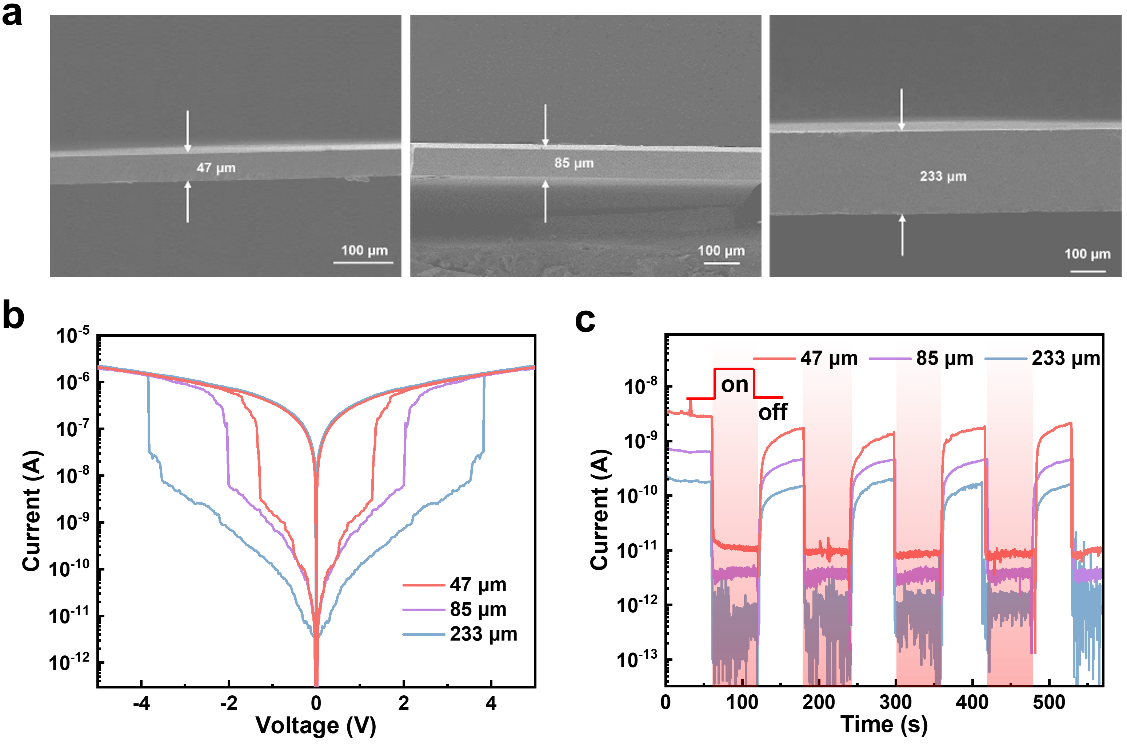


**Fig. S20** **a** Cross-sectional SEM images of the Cs_2_CoCl_4_ SCs with different thicknesses (47, 85 and 233 μm). *I−V* curves (**b)** and *I−t* curves (**c**) of devices with different thicknesses of Cs_2_CoCl_4_ SCs.


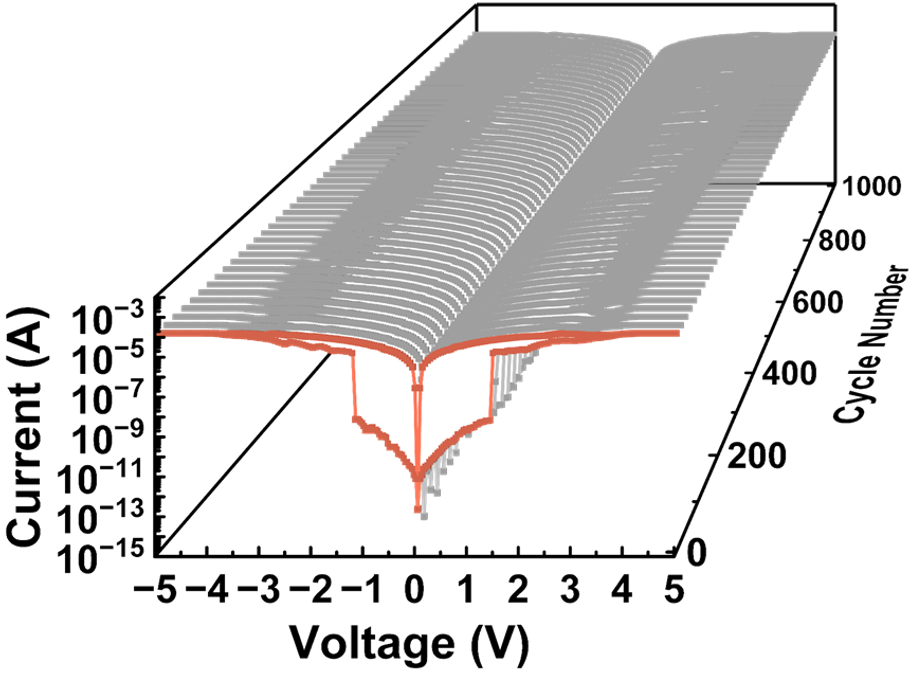


**Fig. S21** Semilogarithmic *I–V* curves of the device for 1000 switching cycles.


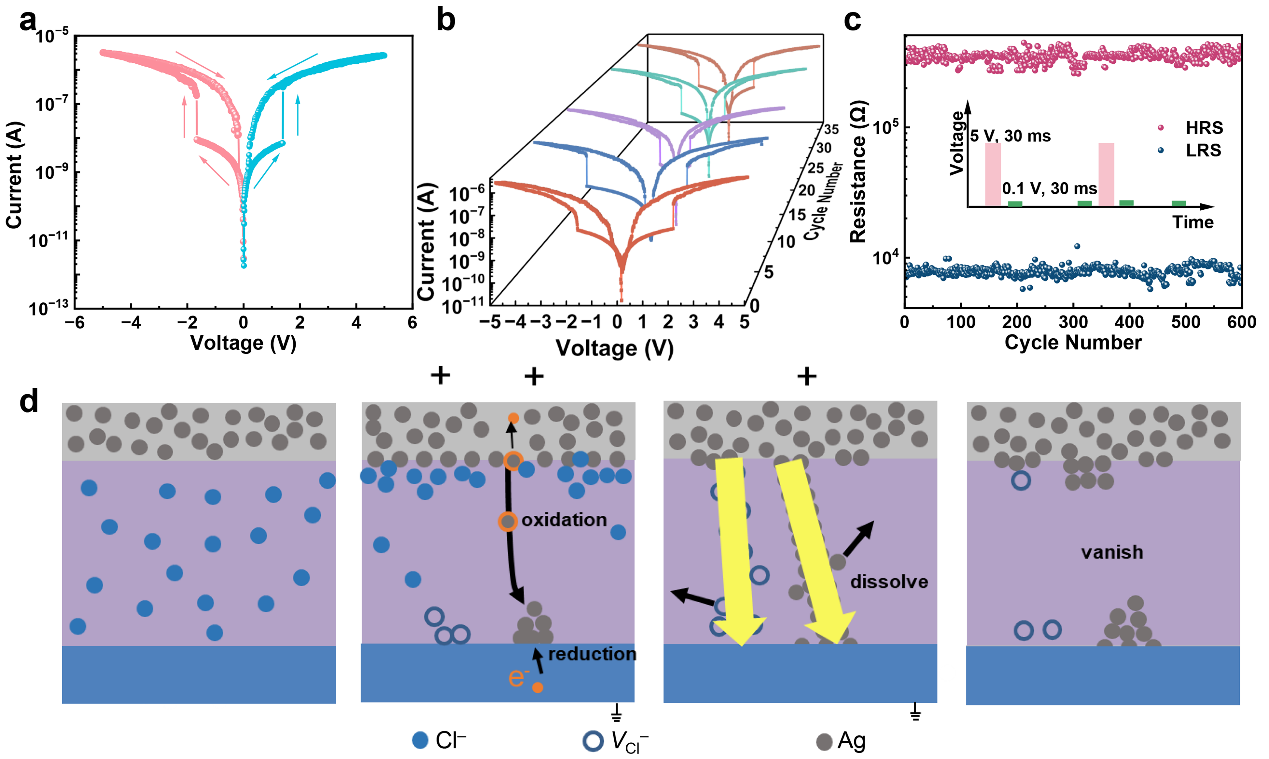


**Fig. S22** **a** Threshold switching in Ag/Cs_2_CoCl_4_/ITO devices. **b** Typical *I–V* characteristics for 50 cycles. **c** Endurance behavior of the devices. **d** Schematic diagram of device resistive switching mechanisms.


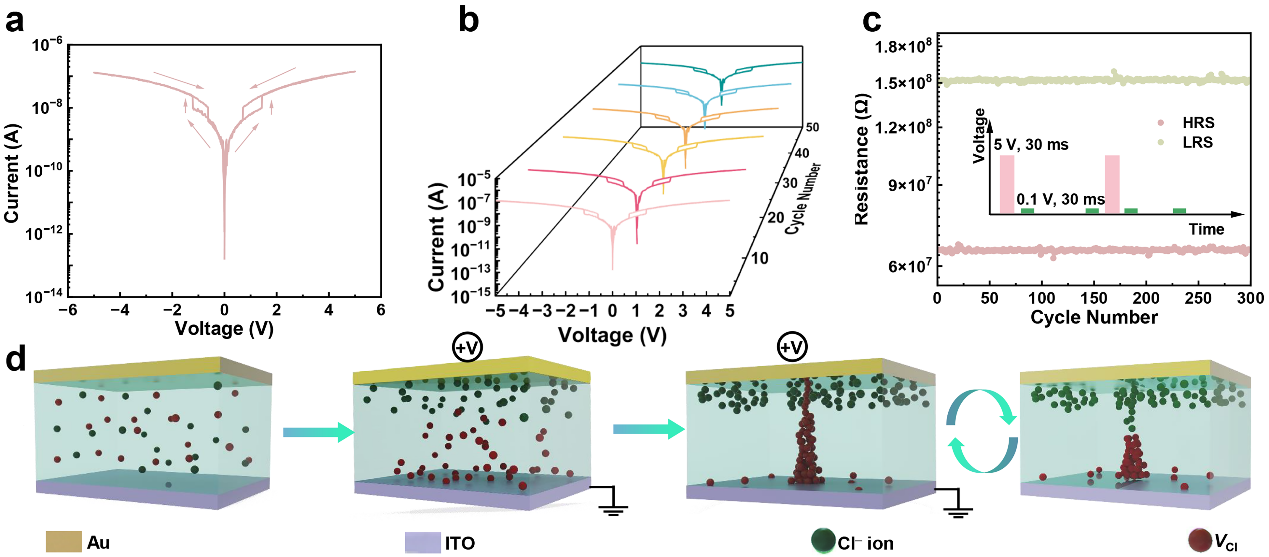


**Fig. S23** **a** Threshold switching in Au/Cs_2_CoCl_4_/ITO devices. **b** Typical *I–V* characteristics for 50 cycles. **c** Endurance behavior of the devices. **d** Schematic diagram of device resistive switching mechanisms.


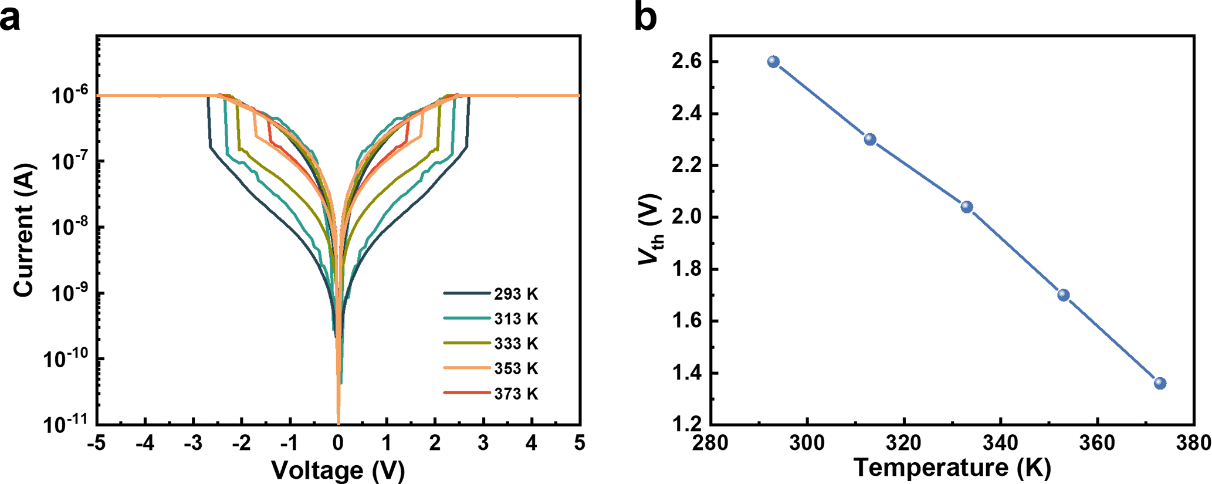


**Fig. S24** Temperature-dependent dark *I–V* curves (**a**) and threshold voltage (**b**) of Au/Cs_2_CoCl_4_/ITO memristor.


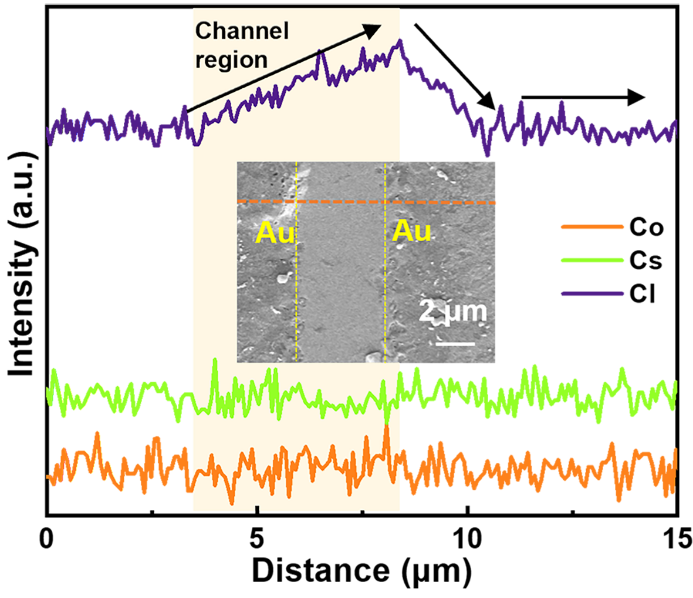


**Fig. S25** EDS line scan profiles of Au electrode-based device with the application of voltage.


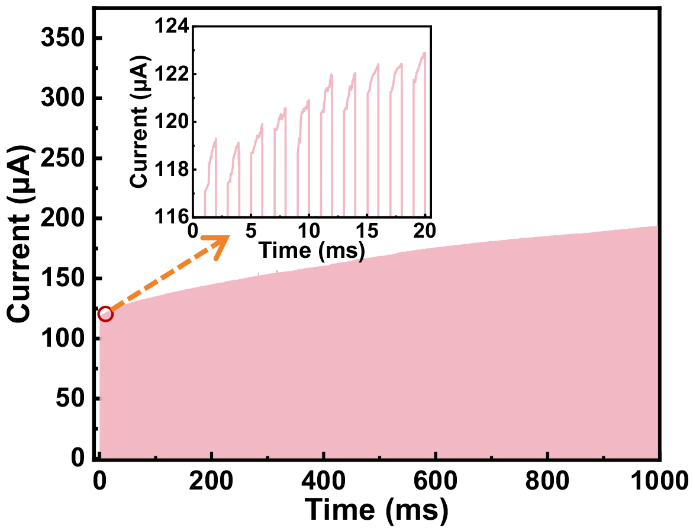


**Fig. S26** PPF behavior stimulated by a pulse train with a pulse amplitude of 5 V, width of 1 ms, and interval of 1 ms.


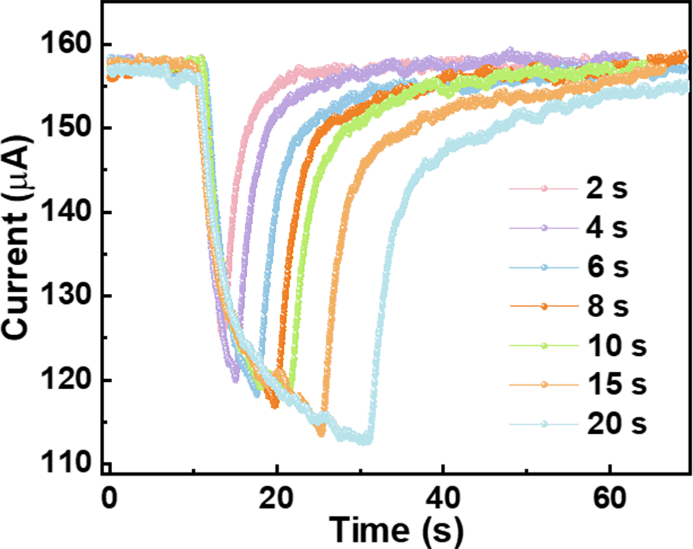


**Fig. S27** IPSC curves generated by a single pulse with varying light durations (2, 4, 6, 8, 10, 15, and 20 s) under a consistent light intensity of 20 mW cm^−2^.


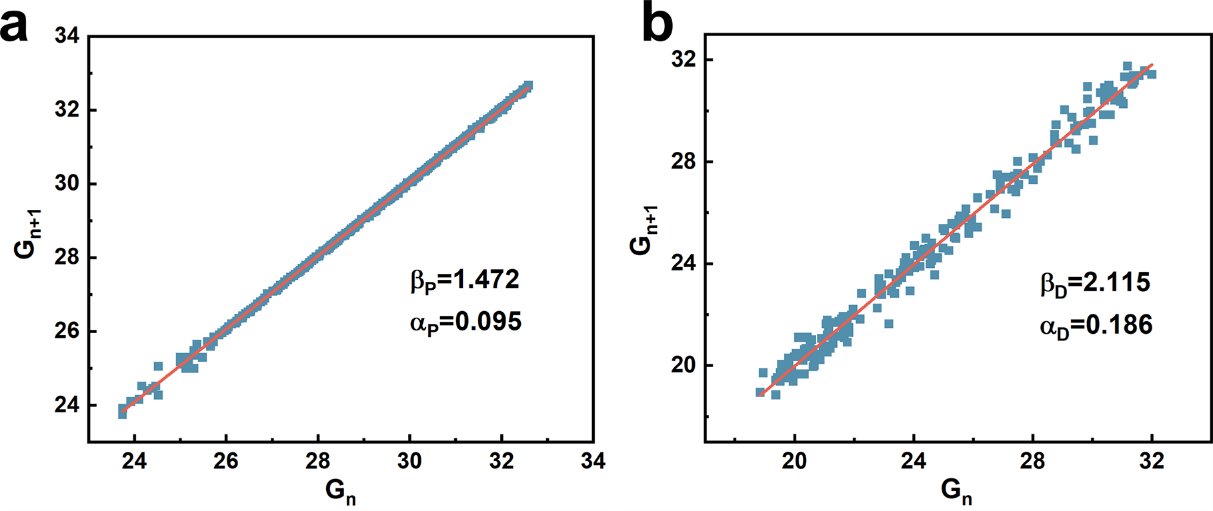


**Fig. S28** Fitting curves made using the experimental data from **a** LTP and **b** LTD.

**Table S1** XRD data of orthorhombic Cs_2_CoCl_4_.

| Compound | Cs_2_CoCl_4_ |
| --- | --- |
| Formula weight (g/mol) | 466.5561 |
| Crystal system | Orthorhombic |
| Space group | Pnam |
| Color | Blue |
| *a* (Å) | 9.7710 |
| *b* (Å) | 12.9730 |
| *c* (Å) | 7.4010 |
| *α* (°) | 90 |
| *β* (°) | 90 |
| *γ* (°) | 90 |
| Volume (Å^3^) | 978.181350 |
| *Z* | 4 |
| ρ_calc_ (g/cm^3^) | 3.30 |

**Table S2** Atomic positions of orthorhombic Cs_2_CoCl_4_.

| Lable | x | y | z | Occupancy |
| --- | --- | --- | --- | --- |
| Cs0 | 0.25000 | 0.85904 | 0.59827 | 1.00000 |
| Cs1 | 0.25000 | 0.35904 | 0.90173 | 1.00000 |
| Cs2 | 0.75000 | 0.14096 | 0.40173 | 1.00000 |
| Cs3 | 0.75000 | 0.64096 | 0.09827 | 1.00000 |
| Cs4 | 0.25000 | 0.52067 | 0.32485 | 1.00000 |
| Cs5 | 0.25000 | 0.02067 | 0.17515 | 1.00000 |
| Cs6 | 0.75000 | 0.47933 | 0.67514 | 1.00000 |
| Cs7 | 0.75000 | 0.97933 | 0.82486 | 1.00000 |
| Co8 | 0.25000 | 0.26654 | 0.5781 | 1.00000 |
| Co9 | 0.25000 | 0.76653 | 0.9219 | 1.00000 |
| Co10 | 0.75000 | 0.73347 | 0.4219 | 1.00000 |
| Co11 | 0.75000 | 0.23347 | 0.0781 | 1.00000 |
| Cl12 | 0.25000 | 0.49189 | 0.59687 | 1.00000 |
| Cl13 | 0.25000 | 0.99189 | 0.90313 | 1.00000 |
| Cl14 | 0.75000 | 0.50811 | 0.40313 | 1.00000 |
| Cl15 | 0.75000 | 0.00811 | 0.09687 | 1.00000 |
| Cl16 | 0.25000 | 0.19126 | 0.41678 | 1.00000 |
| Cl17 | 0.25000 | 0.69125 | 0.08322 | 1.00000 |
| Cl18 | 0.75000 | 0.80875 | 0.58322 | 1.00000 |
| Cl19 | 0.75000 | 0.30874 | 0.91678 | 1.00000 |
| Cl20 | 0.49345 | 0.17818 | 0.6516 | 1.00000 |
| Cl21 | 0.00655 | 0.67818 | 0.8484 | 1.00000 |
| Cl22 | 0.99345 | 0.82182 | 0.3484 | 1.00000 |
| Cl23 | 0.50655 | 0.32182 | 0.1516 | 1.00000 |
| Cl24 | 0.50655 | 0.82182 | 0.3484 | 1.00000 |
| Cl25 | 0.99345 | 0.32182 | 0.1516 | 1.00000 |
| Cl26 | 0.00655 | 0.17818 | 0.6516 | 1.00000 |
| Cl27 | 0.49345 | 0.67818 | 0.8484 | 1.00000 |

**Table S3** Performance comparison of the photodetectors with NPC effect based on various material systems.

| Material | λ (nm) | Detectivity (Jones) | References |
| --- | --- | --- | --- |
| Cs_3_Bi_2_Br_9_ | White light | −2.51×10^12^ | 1 |
| Cs_3_Bi_2_Cl_9_ | White light | −6.23×10^11^ | 2 |
| PtSe_2_ | 532 | −4.34×10^8^ | 3 |
| Cd_3_As_2_ | 365 | −3×10^11^ | 4 |
| α-GaN | 355 | −2.75×10^8^ | 5 |
| Graphene | 365 | −2.4 5×10^9^ | 6 |
| Ga_2_O_3_ | 365 | −2.52×10^11^ | 7 |
| WSe_2_ | 365 | −1.6×10^13^ | 8 |
| GeS | 405 | −1×10^12^ | 9 |
| Cs_2_CoCl_4_ | 265  360  405  520  650  780 | −2.1×10^12^  −5.1×10^11^  −8.9×10^11^  −2.1×10^12^  −2.8×10^12^  −3.3×10^11^ | This work |

**Table S4.** Summary of resistive switching device performances based on various material systems.

| Material | Switching Ratio | Active Layer Thickness | Transition Voltage (V) | 1/Electric Field Strength (m/V) | Ref. |
| --- | --- | --- | --- | --- | --- |
| NiO | 10^2^ | 75 nm | 1 | 7.5×10^–8^ | 10 |
| As-Te-Ge-Si-N | 1.5×10^1^ | 70 nm | 1.2 | 4.12×10^-8^ | 11 |
| Pt-Fe_2_O_3_ NP | 10^1^ | 80 nm | 2.5 | 3.2×10^–8^ | 12 |
| TiO_2_ | 10^7^ | 4 nm | 0.24 | 1.67×10^–8^ | 13 |
| Ferritin | 10^5^ | 250 nm | 1.3 | 1.92×10^–7^ | 14 |
| Ag-Cu_2_O | 5×10^2^ | 250 nm | 0.6 | 4.17×10^–7^ | 15 |
| Si-As-Te | 10^2^ | 135 nm | 1.5 | 9×10^–8^ | 16 |
| NbO_x_ | 10 | 120 nm | 2.5 | 4.8×10^–8^ | 17 |
| PFO_x_R_y_ | 2×10^5^ | 50 nm | 2.5 | 2×10^–8^ | 18 |
| V_2_O_5_ | 1.2×10^1^ | 80 nm | 0.45 | 1.78×10^–7^ | 19 |
| Ag:Si | 10^7^ | 10 nm | 0.8 | 1.25×10^–8^ | 20 |
| Protein | 10^8^ | 120 nm | 1.7 V | 7×10^–8^ | 21 |
| Ag nanodots/HfO_2_ | 10^8^ | 7.78 nm | 0.25 V | 3.13×10^–8^ | 22 |
| HfTiO | 10^2^ | 20 nm | 1.8 V | 1.1×10^–8^ | 23 |
| Cs_3_Sb_2_Br_9_ | 10^6^ | 10 μm | 2.2 V | 4.5×10^–6^ | 24 |
| Cs_2_CoCl_4_ | 10^6^ | 47 μm | 2.3 V | 2×10^–5^ | This work |

**References**

1. Tailor, N. et al. Observation of negative photoconductivity in lead-free Cs_3_Bi_2_Br_9_ perovskite single crystal. *ACS Photonics* **8**, 2473–2480 (2021).
2. Tailor, N. et al. Dark self-healing-mediated negative photoconductivity of a lead-free Cs_3_Bi_2_Cl_9_ perovskite single crystal. *J. Phys. Chem. Lett.* **12**, 2286–2292 (2021).
3. Zhang, H. et al. PtSe_2_ Field-effect phototransistor with positive and negative photoconductivity. *ACS Appl. Electron. Mater.* **4**, 5177–5183 (2021).
4. Park, K. et al. Phase controlled growth of Cd_3_As_2_ nanowires and their negative photoconductivity. *Nano Lett.* **20**, 4939–4946 (2021).
5. Singh, D. et al. Photocurrent polarity switching and enhanced photoresponse in silver nanoparticles decorated α-GaN-based photodetector. *ACS Appl. Electron. Mater.* **5**, 1394–1400 (2021).
6. Shen, J. et al. Multifaceted roles of copper ions in anticancer nanomedicine. *Adv. Opt. Mater.* **11**, 2300410 (2021).
7. Ruan, W. et al. Observation of anomalous negative photoconductivity in Ga_2_O_3_ nanowires: implications for broadening the spectral response of photodetectors. *ACS Appl. Nano Mater.* **6**, 1019–1026 (2021).
8. Zou, Y. et al. High-temperature flexible WSe_2_ photodetectors with ultrahigh photoresponsivity. *Nat. Commun.* **13**, 4372 (2021).
9. Zhao, S. et al. In situ growth of GeS nanowires with sulfur-rich shell for featured negative photoconductivity. *J. Phys. Chem. Lett.* **12**, 3046 (2021).
10. He, L. et al. Memory and threshold resistance switching in ni/nio core–shell nanowires. *Nano Lett.* **11**, 4601–4606 (2021).
11. Lee, M. et al. A plasma-treated chalcogenide switch device for stackable scalable 3D nanoscale memory. *Nat. Commun.* **4**, 2629 (2021).
12. Baek, Y. et al. Tunable threshold resistive switching characteristics of Pt–Fe_2_O_3_ core–shell nanoparticleassembly by space charge effect. *Nanoscale* **5**, 772–779 (2021).
13. Song, J. et al. Threshold selector with high selectivity and steep slope for cross-point memory array. *IEEE Electron Device Lett.* **36**, 681–683 (2021).
14. Zhang, C. et al. Convertible resistive switching characteristics between memory switching and threshold switching in a single ferritin-based memristor. *Chem. Commun.* **52**, 4828–4831 (2021).
15. Song, J. et al. Bidirectional threshold switching in engineered multilayer (Cu_2_O/Ag:Cu_2_O/Cu_2_O) stack for cross-point selector application. *Appl. Phys. Lett.* **107**, 113504 (2021).
16. Lee, J. et al. Threshold switching in Si-As-Te thin film for the selector device of crossbar resistive memory. *Appl. Phys. Lett.* **100**, 123505 (2021).
17. Bae, J. et al. Coexistence of Bi-stable memory and mono-stable threshold resistance switching phenomena in amorphous NbOx films. *Appl. Phys. Lett.* **100**, 062902 (2021).
18. Ling, Q. et al. A dynamic random access memory based on a conjugated copolymer containing electron-donor and -acceptor moieties. *Angew. Chem. Int.* *Ed.* **118**, 3013–3017 (2021).
19. Xue, W. et al. A 1D vanadium dioxide nanochannel constructed via electric‐field‐induced ion transport and its superior metal–insulator transition. *Adv. Mater.* **29**,1702162 (2021)*.*
20. Yoo, J. et al. Threshold switching behavior of Ag-Si based selector device and hydrogen doping effect on its characteristics. *AIP Adv.* **5**, 127221 (2021).
21. Wang, H. et al. Configurable resistive switching between memory and threshold characteristics for protein‐based devices. *Adv. Funct. Mater.* **25**, 3825 (2021).
22. Li, Y. J et al. High‐Uniformity threshold switching HfO_2_ ‐based selectors with patterned ag nanodots*. Adv. Sci.* **7**, 2002251 (2021).
23. Ye, C. et al. Hafnium nanocrystals observed in a HfTiO compound film bring about excellent performance of flexible selectors in memory integration. *Nanoscale* **11**, 20792–20796 (2021).
24. Mao, J. et al. Lead-free monocrystalline perovskite resistive switching device for temporal information processing. *Nano Energy* **71**, 104616 (2021).
